# 4931414P19Rik, a microglia chemoattractant secreted by neural progenitors, modulates neuronal migration during corticogenesis

**DOI:** 10.1242/dev.201574

**Published:** 2023-04-28

**Authors:** Ivan Mestres, Federico Calegari

**Affiliations:** CRTD-Center for Regenerative Therapies Dresden, Technische Universität Dresden, Fetscherstrasse 105, 01307 Dresden, Germany

**Keywords:** Brain development, Neuronal migration, Microglia, Macrophage, Chemoattraction, 4931414P19Rik

## Abstract

Communication between the nervous and immune system is crucial for development, homeostasis and response to injury. Before the onset of neurogenesis, microglia populate the central nervous system, serving as resident immune cells over the course of life. Here, we describe new roles of an uncharacterized transcript upregulated by neurogenic progenitors during mouse corticogenesis: 4931414P19Rik (hereafter named P19). Overexpression of P19 cell-extrinsically inhibited neuronal migration and acted as chemoattractant of microglial cells. Interestingly, effects on neuronal migration were found to result directly from P19 secretion by neural progenitors triggering microglia accumulation within the P19 targeted area. Our findings highlight the crucial role of microglia during brain development and identify P19 as a previously unreported player in the neuro-immune crosstalk.

## Teaser

**Summary:** Secretion of the previously uncharacterized protein 4931414P19Rik by neural progenitors is crucial for mediating neuro-immune crosstalk and for regulating neuronal migration during brain development.

## INTRODUCTION

During mammalian brain development, different types of stem and progenitor cells populate the germinal layers, including apical and basal progenitors. Apical progenitors within the ventricular zone (VZ) expand through proliferative division, and can also give rise to basal progenitors forming the sub-ventricular zone (SVZ). In turn, basal progenitors generate neurons that migrate through the intermediate zone (IZ) to reach their final destination within the cortical plate (CP) (Lui et al., 2011; Taverna et al., 2014). As a result, the balance between proliferative and neurogenic divisions, and the proper migration of newborn neurons, are fundamental during development to establish the cytoarchitecture and size of the adult brain.

In an attempt to reveal novel mechanisms involved in mammalian corticogenesis, our group previously identified a subset of genes that are transitorily up- or downregulated specifically by neurogenic progenitors relative to both proliferative progenitors and newborn neurons (Aprea et al., 2013). The functional implication of some of these transcripts that identify the signature of neurogenic commitment, which are referred to as up- or down-switch genes, was revealed in subsequent studies focusing on various classes of non-coding RNAs, pioneer transcription factors and epigenetic mechanisms (Aprea et al., 2015; Artegiani et al., 2015; Dori et al., 2019, 2020; Noack et al., 2019). Also among the up-switch genes, we found an uncharacterized transcript that, until now, was identified with an annotation number: 4931414P19Rik (human homologue C14orf93; henceforth referred to as P19). To date, we can find only two reports on P19 that associated mutations in its locus with thyroid function and cancer (Liu et al., 2017; Yu et al., 2017). However, the biological implication of this association with thyroid cancer, or underlying molecular mechanism, was not explored. In addition, the role of P19 in tissues other than the thyroid, and particularly the developing brain, was not investigated, despite the fact that genome-wide association studies have linked P19 mutations to schizophrenia (Lam et al., 2019). This prompted us to characterize the function of P19 during mammalian corticogenesis, where we found an unexpected role for it as a modulator of the crosstalk between neural progenitors and microglia.

Although links between the nervous and the immune system are often neglected in the study of neural stem cell fate and neuronal migration, it is intriguing to note that the onset of neurogenesis coincides with microglia colonization of the brain at mouse embryonic day (E) 9-10 (Ginhoux et al., 2010). Specifically, microglia populate the VZ/SVZ and IZ while initially avoiding the CP (Hattori and Miyata, 2018; Hattori et al., 2020), which is colonized only later after neurogenesis is completed (∼E18) (Cunningham et al., 2013). Derived from a precursor in common with microglia, non-parenchymal macrophages also reside within specific niches of the brain under homeostatic conditions, including the choroid plexus, meninges and perivascular space (Utz et al., 2020). Finally, upon injury or disease, monocyte-derived macrophages can infiltrate the brain from the blood in a process referred to as neuroinflammation (Han et al., 2021). Importantly, perturbations of the immune system during gestation are causally linked to several neurodevelopmental disorders (Han et al., 2021). This raises several fundamental questions pertaining to which signals attract brain microglia and/or macrophages to populate specific layers of the cortex and whether their accumulation plays any role in neural cell fate specification and/or migration of newborn neurons. Although some attempts were made to address the role of microglia in corticogenesis (Arnò et al., 2014; Cunningham et al., 2013; Hattori and Miyata, 2018; Hattori et al., 2020), the answer to these questions remains elusive.

Here, we identify P19 as a novel cell-extrinsic chemoattractant of microglia that promotes their accumulation within the germinal zones. In turn, we show that P19 secretion by neural progenitors is crucial for mediating neuro-immune crosstalk and for regulating neuronal migration during brain development.

## RESULTS

### P19 cell-extrinsically controls neuronal migration

Our group previously identified P19 as an up-switch gene, i.e. a transcript identifying the signature of neurogenic commitment whose expression during corticogenesis is the highest in progenitors undergoing neurogenic division relative to both proliferative progenitors and newborn neurons (Aprea et al., 2013) (Fig. S1A,B). Specifically, P19 was not only among the top 50% most expressed transcripts in the embryonic brain but was also, when analyzing proliferative versus neurogenic progenitors and neurons of the E14 mouse cortical wall, upregulated approximately twofold in neurogenic progenitors relative to the other two cell types (normalized reads: 241±15, 392±17 and 242±7, proliferative progenitors, neurogenic progenitors and neurons, respectively; *P*<0.05). To corroborate our previous findings in the E14 mouse cortex, we took advantage of single-cell transcriptome analyses (Cao et al., 2019; Di Bella et al., 2021; Telley et al., 2019) confirming ∼1.5-fold higher expression of P19 in neurogenic progenitors compared with radial glial cells and postmitotic neurons when evaluated between E9 and E13. Moreover, by combining fluorescent immunohistochemistry with *in situ* hybridization in E14 brain sections, we additionally validated that Tbr2^+^ neurogenic progenitors within the SVZ displayed nearly threefold higher levels of P19 expression relative to both proliferating progenitors and neurons (Fig. S1C,D).

**Fig. 1.**
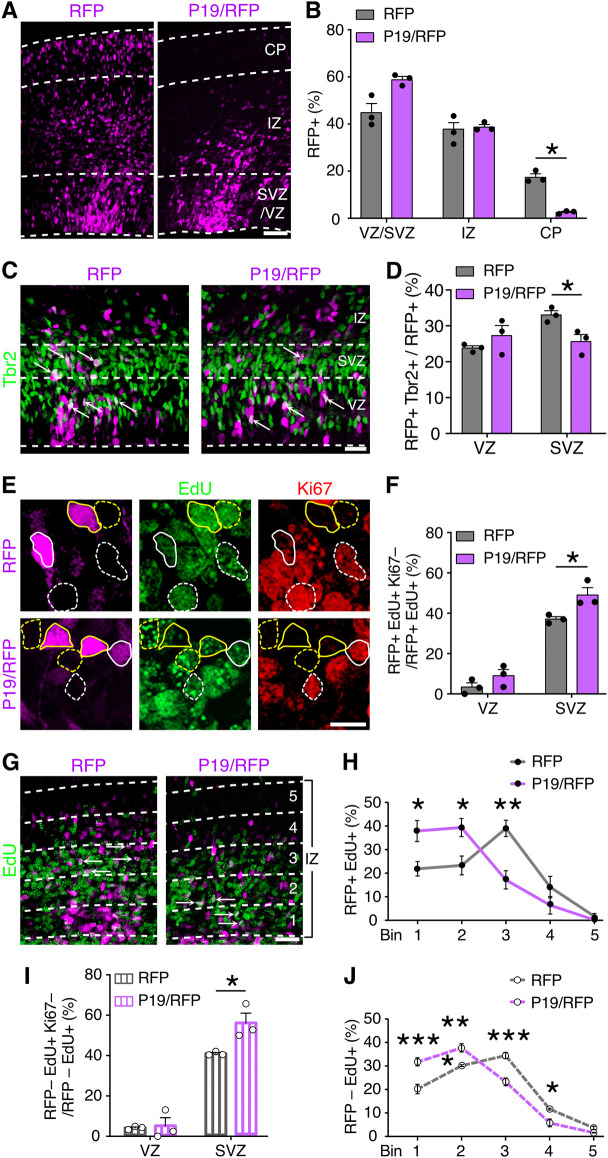
**P19 is an up-switch gene involved in corticogenesis.** (A,C,E,G) Fluorescence images of coronal sections of E15 brains 2 days after electroporation with control RFP or P19/RFP plasmids (pseudo-colored in magenta) and immunolabeled with markers of basal progenitors, proliferation and S-phase (Tbr2, Ki67 and EdU, respectively, as indicated). Dashed lines indicate the borders of the ventricular/subventricular zones (VZ/SVZ), intermediate zone (IZ) and cortical plate (CP) (A,C), or bins within the IZ (G). Outlines in E indicate examples of cells positive (continuous) or negative (dashed) for RFP; and Ki67^+^ or Ki67^–^ cells (white and yellow lines, respectively). Lower magnifications of E are shown in Fig. S1K. (B,D,F,H-J) Percentages of cells quantified from the respective panels (A,C,E,G), considering RFP^+^ (B,D,F,H) or adjacent RFP^–^ (I,J) cells. Data are mean±s.e.m. with individual data points (B,D,F,I) or mean±s.e.m. (H,J) Arrows in C and G indicate double-positive cells. Two-way ANOVA and Bonferroni's post-hoc test were used to assess significance (*P<0.05, **P<0.01, ***P<0.001). Scale bars: 50 µm in A,G; 25 µm in C; 10 µm in E.

To investigate the function of P19, we next performed *in utero* electroporation of the cortical wall at E13 using plasmids encoding either a red fluorescent protein (RFP) alone as a control, or P19 together with RFP (P19/RFP) under two independent constitutive promoters. This approach resulted in a threefold overexpression of P19 within targeted cells (RFP, 1.1±0.27 versus P19/RFP, 2.9±0.07; *P*<0.01), as assessed by qRT-PCR from fluorescence-activated cell sorted RFP^+^ cells 2 days after electroporation. To evaluate the effects of such P19 overexpression on corticogenesis, brains were collected 2 days after electroporation and processed for immunohistochemistry. At E15, we observed that the largest fraction of RFP^+^ cells electroporated with either control or P19/RFP plasmids were retained within the proliferative areas (VZ/SVZ) or migrated into the IZ (Fig. 1A). Although no major difference between control and P19/RFP electroporated brains was observed in the proportion of RFP^+^ cells within the VZ/SVZ or IZ, P19/RFP electroporated brains displayed an almost complete lack of RFP^+^ cells in the CP relative to controls (CP RFP, 17.3±2.2% versus P19/RFP, 2.5±0.5%; *P*<0.05) (Fig. 1A,B). This effect was unlikely to be due to apoptosis, as immunolabeling for caspase 3 showed no difference between brains electroporated with either construct (Fig. S1E,F).

We next sought to examine whether P19 modulated different aspects of neural progenitor cell fate. We started by evaluating the mitotic index within the VZ or SVZ by quantifying the proportion of RFP^+^ transfected cells counterstained with the mitotic marker phospho-histone 3 (PH3), which revealed neither major nor significant differences between control or P19/RFP vectors (Fig. S1G,H). In contrast, we observed a minor, although significant, decrease in the proportion of electroporated cells positive for the basal progenitors marker Tbr2 within the SVZ, but not the VZ, of P19/RFP electroporated brains relative to control (SVZ RFP, 33.0±0.9% versus P19/RFP, 25.6±1.6%; *P*=0.04) (Fig. 1C,D). Additionally, we performed electroporation in the *Btg2*::GFP reporter mouse line as a means to directly identify progenitors (either apical or basal) committed to neurogenic divisions (Haubensak et al., 2004). This experiment showed that, compared with brains transfected with control plasmids, P19 again triggered a decrease in the proportion of *Btg2*::GFP^+^ progenitors specifically within the SVZ, but not the VZ, the magnitude of which was similar to that assessed by Tbr2 (SVZ RFP, 29.0±1.5% versus P19/RFP, 21.6±1.4%; *P*<0.04) (Fig. S1I,J).

The almost complete absence of neurons in the CP upon P19/RFP overexpression was hard to explain solely by a decrease in neurogenic divisions, given that the observed reduction in Tbr2^+^ (basal) and Btg2^+^ (neurogenic) progenitors within the SVZ was minor and barely significant. Alternatively, we argued that a more likely explanation for the lack of RFP^+^ cells in the CP was that P19 impaired the migration of newborn neurons, which would also result in an increase in the proportion of Tbr2^–^ and Btg2^–^ postmitotic neurons retained within the SVZ. We reasoned that this was even more likely considering that, although the proportion of RFP^+^ cells in the IZ of P19/RFP electroporated brains was similar to control, their distribution was clearly biased apically towards the SVZ (Fig. 1A).

To validate a possible increase in postmitotic neurons within the SVZ upon P19/RFP overexpression, we assessed cell cycle exit by means of a single injection of EdU 24 h after electroporation and collecting the brains 24 h thereafter. Immunolabeling with the proliferation marker Ki67 was performed to assess cells that exited the cell cycle within this developmental time but that were still retained within the SVZ. Interestingly, we found that not only did an increased proportion of RFP^+^ cells exit the cell cycle (EdU^+^ Ki67^–^/EdU^+^) in the SVZ of P19/RFP electroporated brains, but also that this increase matched in magnitude (SVZ RFP, 37.0±0.9% versus P19/RFP, 49.0±2.9%; *P*=0.04) (Fig. 1E,F) the decrease in Tbr2^+^ or Btg2^+^ cells described above.

Next, we sought to directly confirm the effects of P19/RFP overexpression on neuronal migration. To this aim, experiments in which mice were administered EdU 24 h before sacrifice (see above) were used as a birth-dating strategy to assess the migration of RFP^+^ EdU^+^ cells by evaluating their distribution across five equally sized bins within the IZ. This analysis revealed that, upon electroporation with control plasmids, twice as many RFP^+^ neurons localized halfway through the IZ (bin 3) relative to any other bin. In contrast, the majority of P19/RFP-transfected cells accumulated nearer the SVZ and were almost exclusively limited to bins 1 and 2 (Fig. 1G,H).

Together, our analyses revealed that the physiological expression of the up-switch gene P19, as previously identified by our group (Aprea et al., 2013), is important for the proper migration of newborn neurons with little, if any, effect in regulating the balance between proliferative and neurogenic divisions.

While extending the previous functional characterizations of up- and down-switch genes by our group (Aprea et al., 2015; Artegiani et al., 2015; Dori et al., 2019, 2020; Noack et al., 2019), in the current analysis of P19 we observed a completely unexpected phenomenon. To our surprise, when performing the analyses described above not only among RFP^+^ targeted cells but also among their neighboring RFP^–^ untransfected cells, the same effects were found. This included both an increased cell cycle exit within the SVZ (Fig. 1I) and decreased neuronal migration (Fig. 1J). Furthermore, we noted that the magnitude of these effects was virtually identical when assessed among RFP^+^ and RFP^–^ cells of the same brains (Fig. 1F versus I and Fig. 1H versus J). In essence, these results indicated that effects of P19 overexpression were cell extrinsic.

### P19 is a secreted chemoattractant of microglia

**Fig. 2.**
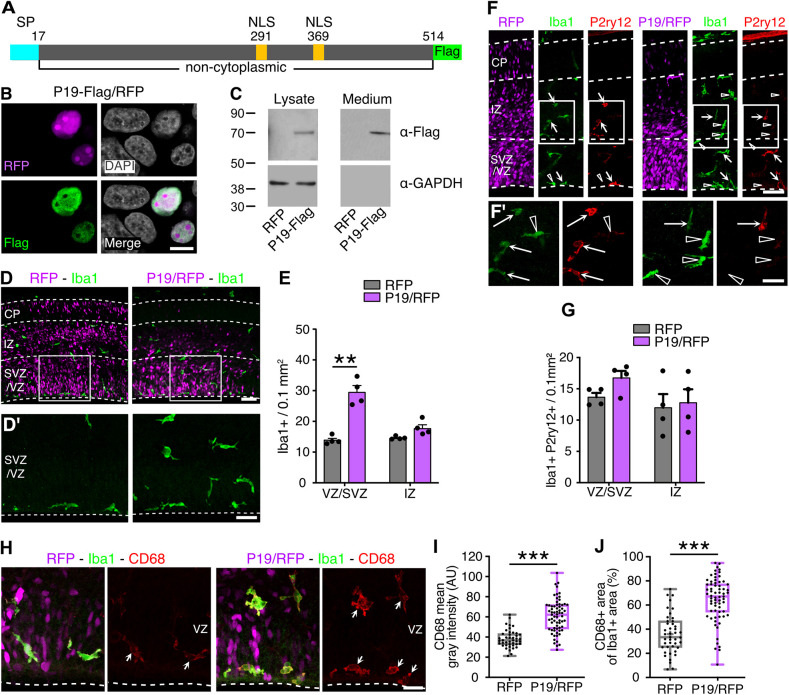
**P19 is a secreted chemoattractant of microglia.** (A) Schematic of P19 primary amino acid sequence, including the predicted signal peptide (SP, light blue) and nuclear localizing signals (NLS, yellow). (B) Fluorescence picture of HEK293 cells transfected with P19-Flag and RFP, and counterstained as indicated. (C) Western blots of cell lysates or culture medium of HEK293 cells transfected with RFP or P19-Flag upon anti-Flag or GAPDH detection. (D,D′,F,F′,H) Fluorescence pictures of coronal brain sections 48 h after electroporation counterstained for Iba1 (green) and the resident microglia marker P2ry12 (red, F) or the lysosomal marker CD68 (red, H). Areas outlined in D and F are magnified in D′ and F′. Arrows indicate Iba1^+^ and P2ry12^+^ or CD68^+^ cells, whereas arrowheads show cells positive for only Iba1. (E,G) Quantification of cell density for the respective markers. (I,J) Mean gray intensity levels (I) or area (J) of CD68 within Iba1^+^ cells. Data are mean±s.e.m. (E,G). The boxes and whiskers indicate 25th to 75th percentiles, and minimum to maximum values, respectively (I,J). Two-way ANOVA and Bonferroni's post-hoc test (E,G) or two-tailed unpaired *t*-test (I,J) were used to assess significance (**P<0.01, ***P<0.001). Scale bars: 10 µm in B; 50 µm in D,F; 25 µm in D′,F′; 20 µm in H.

Given the surprising cell-extrinsic effects of P19, we next sought to inspect features within its sequence that would provide indications about its function and secretion. However, analyses of the primary amino acid sequence by InterPro (Blum et al., 2021) and the secondary structure by HHPred (Zimmermann et al., 2018) failed to reveal any conserved catalytic domain that could be used to deduce a molecular function. Nevertheless, two important aspects of P19 were highlighted. First, predictions by NLS Mapper (Kosugi et al., 2009) and NLStradamus (Nguyen Ba et al., 2009) identified two bipartite nuclear localization signals starting at residues 291 and 369 (Fig. 2A; Table S1). Second, other bioinformatic prediction software (TOPCONS, Phobius, PrediSi and SignalP) (Hiller et al., 2004; Käll et al., 2004; Petersen et al., 2011; Tsirigos et al., 2015) indicated a signal peptide within the first 17 amino acids from the N-terminal and a cleavage site between amino acids 17 and 18, both of which are hallmarks of secreted proteins (Fig. 2A; Table S1). The rest of the P19 sequence was predicted to be only ‘non-cytoplasmic’. We reasoned that although the significance of the predicted nuclear localization signals remained unclear, the presence of an N-terminal signal peptide, cleavage site, lack of predicted transmembrane domains and ‘non-cytoplasmic’ sequence were all consistent with P19 being a soluble protein secreted via vesicular exocytosis.

Next, to confirm the nuclear localization and secretion of P19, we used HEK293 cells transfected with a dual promoter construct and independently co-overexpressing (1) P19 fused to a Flag tag at the C-terminus and (2) a nuclear-localized RFP (P19-Flag/RFP). Subsequent Flag immunolabeling showed that P19 localized to the nucleus (with the exception of nucleoli) (Fig. 2B). To validate whether P19 was also secreted, a similar experiment was performed, but this time by obtaining cell lysates and culture media to assess P19-Flag by western blot analyses. This revealed a single band at 70 kDa in both the cellular and medium fractions (Fig. 2C). The increase between the predicted (58 kDa) and the observed size can be explained by possible post-translational modifications, such as glycosylation, which is typical of secreted proteins. Consistent with bioinformatic predictions, these experiments supported both P19 localization in the nucleus and its secretion.

The finding that P19 is a secreted protein raised the possibility that its cell-extrinsic effects upon *in utero* electroporation are not limited to neural progenitors and newborn neurons, but can also apply to any other cell type of the developing cortex. Although astrocytes and oligodendrocytes first appear at later stages of development (Rowitch and Kriegstein, 2010), at the time of P19/RFP overexpression (E13-15), and specifically within the cortical wall, there are essentially only three cell types in addition to neural progenitors and projection neurons: interneurons, endothelial cells and macrophages, with the last including microglia proper, perivascular macrophages or, potentially, infiltrating monocytes. Hence, to test the effects of P19/RFP overexpression on these cell types, we subjected brain slices electroporated as described above to immunolabeling with antibodies against calbindin (interneurons), CD31 (endothelial cells) and Iba1 (microglia/macrophages).

When analyzing the migratory stream of interneurons that was the closest to the SVZ (Marín et al., 2010), where most electroporated cells localize, we could not find any obvious change in the total number of calbindin^+^ cells per area or in their distribution across three equally sized bins perpendicular to the ventricular surface (to account for tangential, rather than radial, migration of this neuronal population) (Fig. S2A-C). Similarly, when assessing the blood vessels by means of CD31 labelling, none of the parameters considered, including their density, length, diameter and branching, were changed upon P19/RFP overexpression (Fig. S2D-I). Finally, we assessed brain macrophages by immunolabeling with the marker Iba1. This showed that, in agreement with previous reports (Cunningham et al., 2013), Iba1^+^ cells almost exclusively localized within the VZ/SVZ and IZ (Fig. 2D), and that their distribution was homogenous within the RFP^+^ electroporated area or contralateral hemisphere and comparable with that found in naïve non-electroporated brains (not shown). In contrast, remarkably, microglia displayed a greater density within the electroporated area of P19/RFP targeted brains (Fig. 2D,E).

Specifically, in P19/RFP electroporated brains, and particularly within the VZ/SVZ, the number of Iba1^+^ cells more than doubled when compared with brains electroporated with control plasmids (VZ/SVZ RFP, 13.9±1.2 versus P19/RFP, 29.4±4.0 Iba1^+^ cells per 0.1 mm^2^; *P*<0.01). Clearly, macrophages themselves were not targeted by electroporation, given that these cells do not contact the ventricular surface where plasmids were injected and, consistently, no colocalization of RFP with Iba1 was found (e.g. Fig. 2D). In sum, this implied that macrophage accumulation within the targeted area resulted from P19 secretion from neural progenitors.

Given that Iba1 does not discriminate between different classes of macrophages, we next sought to identify distinct cell types by labelling with a combination of molecular markers: Ccr2, CD206 and Lyve1 for non-parenchymal or infiltrating macrophages; and P2ry12 for microglia. We noticed both in control and P19/RFP electroporated brains that Ccr2^+^ macrophages were negligible within the brain areas analyzed and that the few detected were negative for Iba1 (Fig. S2J). In addition, the use of CD206 and Lyve1 revealed non-parenchymal macrophages within the choroid plexus and meninges, but not at the level of the perivascular space (data not shown), which is consistent with reports showing the negligible contribution by this population at the stages of development analyzed (Utz et al., 2020). Altogether, these results exclude the possibility that accumulation of Iba1^+^ cells upon P19/RFP overexpression results from infiltrating monocytes or perivascular macrophages colonizing the brain parenchyma.

Intriguingly, although in control RFP electroporated brains most Iba1^+^ cells were also immunoreactive for the canonical microglial protein P2ry12, only a fraction of Iba1^+^ cells were labelled with P2ry12 upon P19/RFP overexpression (Fig. 2F,G). The downregulation of P2ry12 suggested the possibility that P19 triggered the molecular response of microglia (Haynes et al., 2006; Paolicelli et al., 2022). To validate this, we used the lysosomal marker CD68, which is usually upregulated in microglia undergoing increased phagocytosis and/or migratory behavior (Papageorgiou et al., 2016). Quantification of CD68 signal intensity and area occupied within Iba1^+^ cells was almost doubled after P19/RFP overexpression compared with control RFP electroporations (CD68 mean gray intensity RFP, 38.2±1.3 versus P19/RFP, 60.8±1.9 AU, *P*<0.0001; CD68^+^ area RFP, 35.9±2.4 versus P19/RFP, 64.5+2.2%, *P*<0.0001) (Fig. 2H-J). These observations further support the notion that microglia responded to P19 secretion from neuronal progenitors, triggering their accumulation within the targeted area.

So far, our observations were based on overexpression data. Thus, we next attempted the converse manipulation of P19 knockdown. However, *in utero* electroporation of a shRNA plasmid, validated in HEK293 cells to silence P19 by ∼70%, did not result in any change in the parameters described above (data not shown). These negative results are not surprising, given that electroporation targets only 25-30% of cells within a background of unmanipulated cells that would still express and secrete P19. In a second attempt, we electroporated endoribonuclease-digested small interfering (esi) RNAs triggering RNAi homogeneously within the tissue (Calegari et al., 2002). However, in this case an increase in Iba^+^ cells was observed not only when targeting P19 but also an unspecific sequence (Luciferase; data not shown). This result suggests that caution is needed when interpreting data derived from esiRNAs gene silencing in the developing brain due to potential nonspecific responses by immune cells. Although neither of the two RNAi methods provided conclusive results, we continued our study focusing on overexpression, deferring to future studies the generation of P19 knockout mice.

### P19 increases microglia speed and migration from neighboring areas

**Fig. 3.**
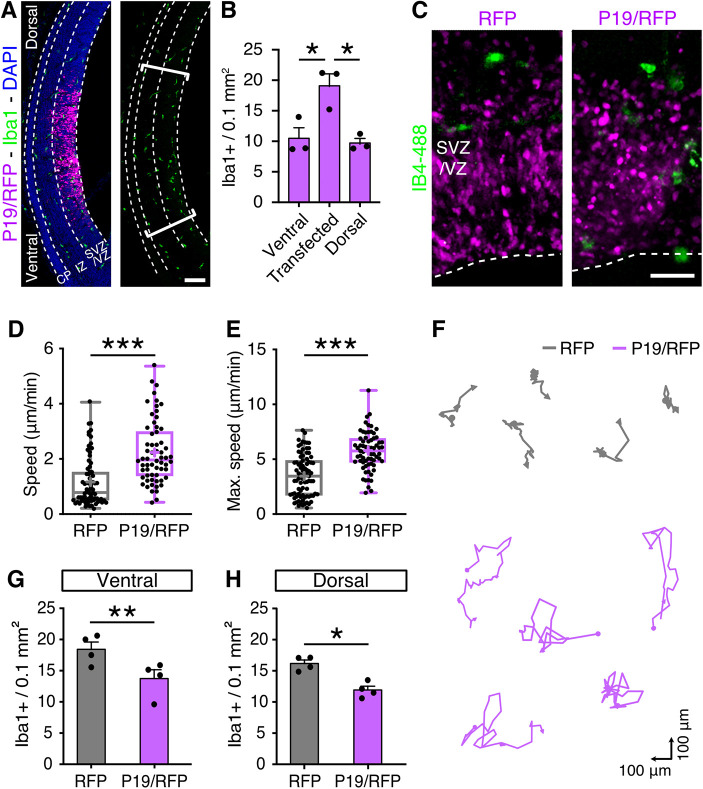
**P19 increases microglia migration.** (A) Coronal brain sections 24 h after electroporation counterstained for Iba1; brackets delimit the electroporated area. (B) Quantification of Iba1^+^ cell density. (C-F) Fluorescence pictures (C), quantifications (D,E) and representative trajectories (F) of IB4-488 labelled microglia (green) obtained by time-lapse microscopy to calculate average and maximum speed (as indicated). (G,H) Iba^+^ cell density at adjacent RFP^–^ areas ventral (G) or dorsal (H) to the electroporated areas. Data are mean±s.e.m. (B,G,H). The boxes and whiskers indicate 25th to 75th percentiles, and minimum to maximum values, respectively (D,E). One-way ANOVA and Tukey's post-hoc test (B) or two-tailed unpaired *t*-test (D,E,G,H) were used to assess significance (*P<0.05, **P<0.01, ***P<0.001). Scale bars: 100 µm in A; 50 µm in C.

Intrigued by our findings, and knowing that embryonic microglia respond quickly upon local environmental changes (Prinz et al., 2019), we investigated whether microglia accumulation upon P19 overexpression was also detected 24 h, rather than 48 h, after electroporation, which is the minimum time necessary to reliably identify the RFP^+^ targeted area. In fact, a greater density of microglia was observed also at this shorter timepoint, with twice as many Iba1^+^ cells in the RFP^+^ P19/RFP-overexpressing area relative to the adjacent RFP^–^ area of the same brains (Fig. 3A,B). We found this increase in microglia accumulation 24 h after electroporation remarkable considering that several hours are necessary for P19 to be expressed, translated and secreted and to accumulate in the extracellular space to the levels needed to trigger a microglia response.

Next, we investigated whether P19 overexpression in neural progenitors triggered changes in other key features that characterize microglia by, specifically, assessing their (1) morphology, (2) proliferation as well as (3) phagocytosis, and (4) migratory behavior.

First, throughout prenatal development, microglia transition from an amoeboid to a branched morphology. Amoeboid microglia present fewer processes and cellular extensions that are shorter than their soma, while, conversely, branched microglia exhibit longer cellular processes and are often ramified (Swinnen et al., 2013). Such co-existence of amoeboid and branched microglia was reflected in brain sections upon electroporation but with no change in their proportions between control or P19/RFP targeted brains (Fig. S3A,B).

Second, to investigate whether accumulation of microglia upon P19/RFP overexpression was due to their increased proliferation, we treated mice with a single injection of EdU 1 day after electroporation at E13 and harvested their brains 24 h thereafter at E15. Similar to previous reports (Arnò et al., 2014; Swinnen et al., 2013), ∼25% of Iba1^+^ microglia were found to be proliferating (EdU^+^) but, again, no difference was found between control and P19/RFP targeted brains (Fig. S3C,D).

Third, microglia can phagocytose neural progenitor cells during development (Cunningham et al., 2013) and display a highly dynamic migratory behavior (Hattori and Miyata, 2018; Swinnen et al., 2013). To assess both parameters, organotypic slice cultures were prepared 24 h after *in utero* electroporation at E13 and treated with isolectin B4 coupled to Alexa Flour 488, which allows direct visualization of microglia (Dailey et al., 2013) (Fig. 3C). Next, dual color time-lapse microscopy was performed over the course of an additional 12 h to evaluate the interaction between transfected progenitors (RFP^+^) and microglia (Alexa-488^+^). Similar to previous studies (Cunningham et al., 2013), phagocytosis events were scored when red and green signals colocalized for more than 45 min and were followed by fragmentation of the RFP signal. Using this approach, no difference was observed in phagocytosis by microglia between brains electroporated with control or P19/RFP vectors (RFP, 6.75±1.4 versus P19/RFP, 8.37±1.7 phagocytosis events in 12 h; *P*=0.48, two-tailed unpaired *t*-test).

Finally, having excluded the effects of P19/RFP overexpression on microglia morphology, proliferation and phagocytosis, the same time-lapse imaging was used to assess their migration. Whereas in control targeted slices, comparable with previous reports (Swinnen et al., 2013), microglia migrated at a speed of 1.16±0.1 µm/min, remarkably, microglia were twice as fast, 2.23±0.1 µm/min (*P*<0.001), in the targeted area of P19/RFP electroporated brains (Fig. 3D). This faster migration of microglia did not seem to result from shorter pause intervals between displacements (defined as the time spent at a speed lower than 10% of the mean of control) (pause intervals RFP, 25.6±3.8 min versus P19/RFP, 17.6±4.9 min; *P*=0.2, two-tailed unpaired *t*-test). Rather, the increased average speed of microglia upon P19/RFP overexpression resulted from a higher maximal speed that almost doubled from 3.50±0.2 to 5.73±0.2 µm/min (*P*<0.001) (Fig. 3E). Consistent with a higher migration speed, plotting individual cell trajectories revealed that microglia covered a larger surveillance area in P19/RFP transfected brains compared with control (Fig. 3F). Our time-lapse imaging, together with our previous quantification of EdU incorporation, suggested that microglia accumulated into the transfected area by migration from neighboring regions rather than by local proliferation. To verify this, we evaluated adjacent RFP^–^ areas, both ventral and dorsal to the electroporated site, and indeed found a reduced number of Iba1^+^ cells compared with similar areas of control RFP targeted brains (Fig. 3G,H). Altogether, these results showed that P19 functions as a chemoattractant of microglia, and that its expression and secretion by neural progenitors results in their accumulation by means of increased migration.

### Microglia accumulation and impaired neuronal migration are causally linked

So far, our analyses show that P19 (1) is primarily expressed by basal neurogenic progenitors of the VZ/SVZ, (2) is a secreted molecule whose overexpression cell-extrinsically impairs neuronal migration, and (3) act as a chemoattractant of microglia, promoting their accumulation and migratory behavior. Together, these observations raise several important questions. First, is P19 secretion per se a direct cause of the phenotypes observed or, alternatively, do these phenotypes arise from secondary effects that electroporated progenitors trigger in response to their P19 overexpression? Second, as the CP is devoid of microglia at the stages of development being considered, can P19 anticipate colonization of this layer when overexpression is performed at the level of the pial, rather than apical, boundary of the cortical wall? And third, are the observed impairments in neuronal migration and accumulation of microglia causally linked rather than two independent effects?

**Fig. 4.**
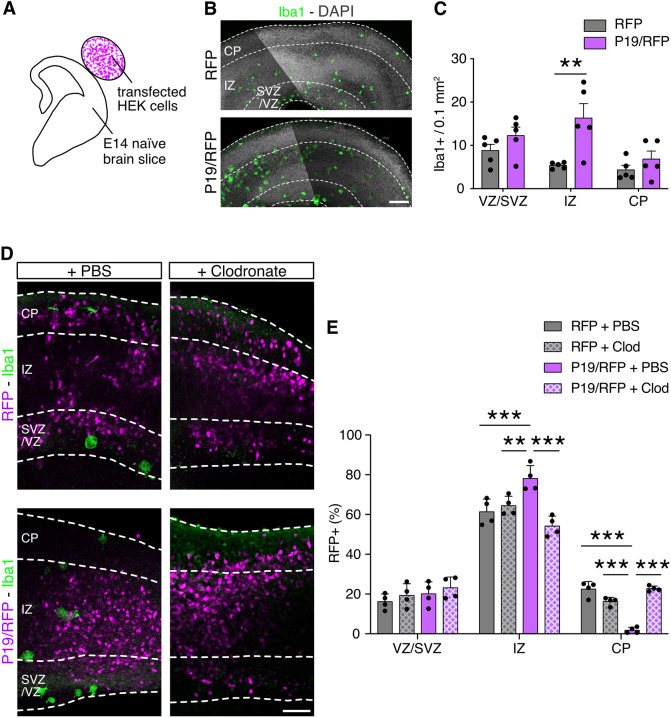
**Depletion of microglia rescues P19-triggered impairment in neuronal migration.** (A) Drawing of brain slice co-culture with transfected HEK293 cells embedded in a collagen drop. (B,C) Fluorescence images (B) of brain slices exposed to HEK293 cells transfected either with RFP or P19/RFP, as depicted in A, followed by immunolabeling and quantification (C) of Iba1^+^ microglia. (D,E) Fluorescent images (D) and quantification (E) of electroporated brain slices incubated with liposomes containing either PBS or clodronate. Data are mean±s.e.m. with individual data points. Two-way ANOVA and Bonferroni's post-hoc test were used to assess significance (**P<0.01, ***P<0.001). Scale bars: 100 µm in B; 50 µm in D.

To address the first two questions, we sought to provide the developing cortex with an ectopic source of P19 but this time using a heterologous cell type instead of endogenous neural progenitors. To achieve this, HEK293 cells were transfected with a control or P19/RFP-overexpressing plasmid and embedded into a collagen drop. Adapting our use of *ex vivo* slice cultures, the HEK293-containing collagen drop was then placed adjacent to the pial surface of E14 brain slices in a similar medio-lateral location to the one usually targeted by electroporation (Fig. 4A). After 24 h of culture, we evaluated the number of microglia by immunolabeling for Iba1 and found that, similar to experiments *in vivo* (Fig. 2D), the proximity to P19/RFP-transfected cells triggered an increased density of microglia in the IZ relative to that observed when using HEK293 cells transfected with control vectors (Fig. 4B,C) (IZ RFP, 5.3±0.2 versus P19/RFP, 16.3±2.9 cells per 0.1 mm^2^; *P*<0.01). Notably, however, because in this experiment the source of P19 was from the pial surface, accumulation of microglia was observed at the basal boundary of the IZ instead of its apical boundary with the SVZ (Fig. 4B). This implies that microglia accumulation is independent not only of the cell type releasing P19 but also of the topological source of its origin. Despite this, we observed that the higher density of microglia within the IZ did not result in their increased invasion of the CP (Fig. 4C), suggesting that P19 alone is not potent enough to override the mechanisms preventing microglia colonization of this cortical layer at this developmental stage. Additionally, although some microglia are known to arise in the CP of cultured *ex vivo* brain slices (Hattori et al., 2020; Swinnen et al., 2013), which are never observed in physiological conditions, their abundance is not influenced by P19.

To address the third question, we investigated whether P19-dependent accumulation of microglia was necessary to inhibit neuronal migration. To achieve this, P19/RFP was overexpressed as described above, but this time microglia accumulation was prevented by exposing brain slices to liposomes loaded with clodronate, which are selectively engulfed by microglia, inducing their cell-specific death (Kumamaru et al., 2012). When assessing slices exposed to PBS-loaded liposomes, and recapitulating previous results *in vivo* (Fig. 1A), we noticed that, compared with electroporation with control plasmids, P19/RFP-targeted slices contained fewer RFP^+^ cells in the CP (CP RFP+PBS, 22.5±1.6 versus P19/RFP+PBS, 1.7±0.6%; *P*<0.001); these cells instead accumulated in the IZ (Fig. 4D,E). In contrast, when assessing the distribution of RFP^+^ cells in slices treated with clodronate-loaded liposomes, the P19-driven impairment in neuronal migration was not observed and the distribution of RFP^+^ cells was undistinguishable from control electroporations (Fig. 4D,E) (CP RFP+clodronate, 16.3±0.8% versus P19/RFP+clodronate, 22.8±4.2%; *P*=0.38). In essence, ablation of microglia completely rescued the P19-triggered effect on neuronal migration. In turn, this suggested that although ablation of microglia in a control background was not sufficient to hinder neuronal migration, the converse accumulation of microglia upon P19/RFP overexpression was necessary to impair neuronal migration.

### Delayed migration triggered defects in cortical layering

**Fig. 5.**
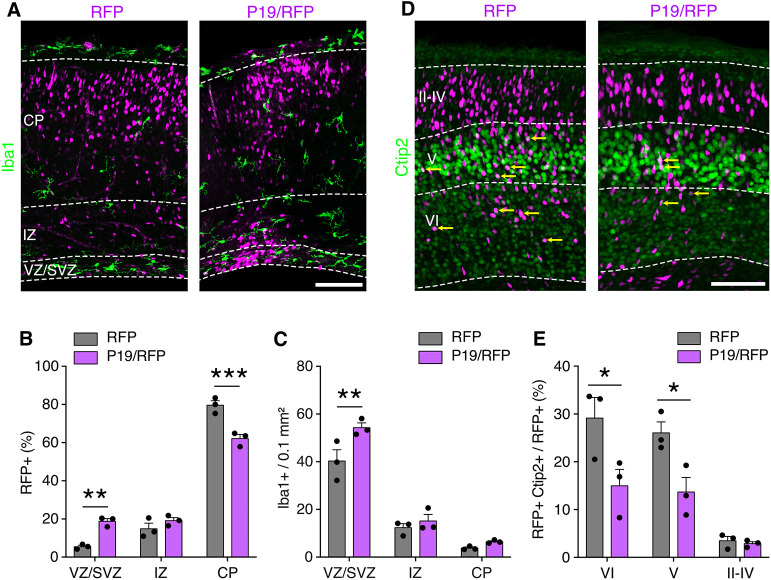
**Delayed migration after P19 overexpression impaired cortical layering.** (A,D) Fluorescence images of brain slices 5 days after electroporation at E13 counterstained for Iba1 (A) or Ctip2 (D). Yellow arrows indicate RFP^+^ and Ctip2^+^ cells. (B,C,E) Percentage of transfected cells (B), density of Iba1^+^ cells (C) or percentage of transfected Ctip2^+^ cells (E). Data are mean±s.e.m. with individual data points. Two-way ANOVA and Bonferroni's post-hoc test were used to assess significance (*P<0.05, **P<0.01, ***P<0.001). Scale bars: 100 µm.

To evaluate whether the phenotypes observed upon P19/RFP overexpression persisted at later stages of development, we decided to analyze the brains at E18, i.e. 5 days instead of 2 days after electroporation at E13. We observed that, compared with the earlier time-point analyzed (E15), P19/RFP overexpression increased the magnitude of cells accumulated within the germinal zones (VZ/SVZ RFP, 5.4±0.5% versus P19/RFP, 18.7±1.1%; *P*=0.0015) at the expense of their migration into the CP (RFP, 79.6±1.9% versus P19/RFP, 63.5±3.1%; *P*=0.0001) (Fig. 5A,B). Similarly, microglia density remained higher in the VZ/SVZ of P19/RFP electroporated brain slices (RFP, 40.2±3.8 versus P19/RFP, 4.3±1.6 Iba1^+^ cells per 0.1 mm^2^; *P*<0.005) (Fig. 5A,C), although to a lower degree compared to E15 brains, which is consistent with the fact that electroporation is a transient overexpression system. At this time-point (E18), the cortex starts to acquire a layered structure, and neurons define their subtype identity. In agreement with the finding that early-born neuron migration is delayed, fewer P19/RFP^+^ neurons were immunolabeled with the deep-layer marker Ctip2 (Fig. 5D,E). Altogether, these results suggest that although the effects on microglia accumulation were attenuated over time, their influence on neuronal migration, and accordingly cellular identity and cortical layering, was not compensated for at later stages of development.

## DISCUSSION

Microglia, non-parenchymal macrophages and infiltrating monocytes were traditionally studied in the context of the adult nervous system, where they engulf apoptotic cells or pathogens, and modulate the inflammatory response (Prinz et al., 2019). However, the distinct function of these three macrophage cell types remains elusive (Utz et al., 2020). More recently, tissue resident microglia were also suggested to execute more sophisticated functions, including the regulation of synapse remodeling and plasticity, and, as a result, cognitive performance (Favuzzi et al., 2021; Nguyen et al., 2020; Roy et al., 2022). In contrast, a role for microglia in controlling neural stem cell fate and neuronal migration during brain development has received much less attention. In this work, we provide the first description of the role of P19 in brain development and in doing so reveal previously unreported insights into the neuro-immune crosstalk during corticogenesis. Although our study could not rule out more-subtle effects of electroporation on microglia identity and additional responses, an increase in microglia density was observed only upon P19/RFP, but not RFP control, overexpression. These results point to P19 as a previously unreported factor secreted by neural progenitors that acts as a chemoattractant of microglia and ultimately influences neuronal migration.

Intriguingly, in our study, P19 secretion selectively acted at the level of microglia without any evident effect on other types of macrophages, including non-parenchymal and infiltrating monocytes. The nature of this selectivity remains unclear. On the one hand, perivascular macrophages within the P19 targeted area of the cortex are few at the stages of development analyzed (Utz et al., 2020), raising the possibility that their accumulation could not be revealed by our study. On the other hand, infiltration of monocytes and their maturation into macrophages is a relatively slow process that requires several days (Ajami et al., 2011) and is unlikely to be observed within the few days after electroporation. However, the possibility remains that P19 is a microglia-specific chemoattractant, raising questions about the significance of its upregulation and secretion specifically by neurogenic progenitors within the SVZ. In this context, it is worth noting that our use of *in utero* electroporation increased P19 expression by threefold within targeted cells, which in turn represent about 25-30% of all apical progenitors within the VZ. In essence, this implies that our approach overall resulted in about a doubling of secreted P19 within the VZ, i.e. mimicking in magnitude the twofold increase in P19 expression in the SVZ that physiologically occurs in the transition between apical and basal progenitors.

Few studies have addressed the mechanisms instructing microglia to colonize the central nervous system during development. Intriguingly, microglia colonization and neurogenic commitment closely coincide during corticogenesis (Ginhoux et al., 2010), raising the possibility that factors released by neurogenic progenitors may act as chemoattractants of microglia, guiding their migration into the brain parenchyma. Consistent with this hypothesis, at least one factor was previously reported to be secreted by neural progenitors and to attract microglia: Cxcl12 (C-X-C motif chemokine ligand 12) (Arnò et al., 2014). Here, we report a second such factor, P19, that selectively promotes the migration of microglia. Notably, while inspecting our previous catalogue of switch genes characterizing neurogenic commitment (Aprea et al., 2013), we found that both Cxcl12 and P19 are on-switch genes. Such a common feature is likely not coincidental considering that on-switch genes are an under-represented class of transcripts consisting of fewer than 1% of the transcriptome of neurogenic progenitors (Aprea et al., 2013). Although not sufficient to override the signals preventing the colonization of the CP, it is tempting to speculate that P19, and probably other on-switch genes expressed and secreted by neurogenic progenitors, are key triggers of microglia colonization of the brain.

The role of the neuro-immune crosstalk during development is receiving increasing attention but only a few studies have addressed whether microglia or other macrophage types can influence the abundance and differentiation of neural progenitors as well as the specification of newborn neurons or their connectivity (Cunningham et al., 2013; Hattori and Miyata, 2018; Hattori et al., 2020; Rosin et al., 2021; Squarzoni et al., 2014). To the best of our knowledge, studies on a direct role of microglia in regulating neuronal migration are lacking. In this context, it is interesting to observe that the same on-switch gene, *Cxcl12*, that was found to increase the density of microglia during development (Arnò et al., 2014) has also been shown, in independent studies, to influence tangential migration of interneurons (Arnò et al., 2014; Borrell and Marín, 2006; Li et al., 2008). Although a causal relationship linking microglia accumulation and neuronal migration by Cxcl12 is lacking, here we find that the accumulation of microglia caused by P19 is necessary to impair neuronal migration. In turn, our study highlights a novel key player of the neuro-immune crosstalk that is important for proper corticogenesis.

On a final note, it is worth remembering that our study additionally showed that P19 can localize to the nucleus, giving it potential to act as more than a secreted molecule. Assessing the functional implication of additional potential function(s) of P19 should be addressed in future studies.

## MATERIALS AND METHODS

### Bioinformatic analyses

The primary amino acid sequence of mouse P19 (UniProt ID: Q8K2W9, 541 residues) was used to infer catalytic domains by InterPro (https://www.ebi.ac.uk/interpro/) and HHPred (https://toolkit.tuebingen.mpg.de/tools/hhpred). In addition, NLS Mapper (http://nls-mapper.iab.keio.ac.jp/) and NLStradamus (http://www.moseslab.csb.utoronto.ca/NLStradamus/) were used to predict nuclear localizing signals. For reference, peptide sequences with a score of 1 and 2 are predicted to localize only to the cytoplasm; scores 3-5 indicate localization in both the cytoplasm and the nucleus; scores 6 and 7 indicate partial nuclear localization; and scores 8-10 predict a mainly nuclear distribution. Additionally, TOPCONS (https://topcons.net/pred/) and Phobius (https://phobius.sbc.su.se/) were used to infer signal peptides; and PrediSi (http://www.predisi.de/index.html) and SignalP (https://services.healthtech.dtu.dk/service.php?SignalP-4.1) to infer cleavage sites in the primary amino acid sequence. Algorithms were run using default settings for eukaryotes.

### Cloning

A cDNA library was constructed using RNA extracted from embryonic mouse cortex (E14) and used as a template to clone the 4931414P19Rik gene-coding sequence. To generate a P19 clone C-terminally fused with a Flag tag (P19-Flag), we employed the following primers: forward, 5′-CAACTATGTCCTTTAGTGCCA-3′; reverse, 5′-GGAAAAGGATGAATACACTCTAGACTACAAAGACGATGACGACAAGTAG-3′. The reverse primer contained the sequence of the Flag tag C-terminally fused to P19. Amplified fragments were then inserted into a pDSV-RFP backbone as previously described (Aprea et al., 2013; Artegiani et al., 2015), and validated by sequencing (Eurofins). The expression of both P19 and RFP was under independent simian virus 40 (SV40) promoters. For the shRNAs, either the ultramer 5′-GTTGACAGTGAGCGCAGGAATTATAATGCTTATCTATAGTGAAGCCACAGATGTATAGATAAGCATTATAATTCCTATGCCTACTGCCTCGGA-3′ targeting Luciferase or the ultramer 5′-TGCTGTTGACAGTGAGCGAGGGCCAACAATGAGTTGTTAATAGTGAAGCCACAGATGTATTAACAACTCATTGTTGGCCCGTGCCTACTGCCTCGGA-3′ targeting P19 was inserted downstream of the GFP sequence under the spleen-focus forming promoter (SFFV) into the mir-E vector (Fellmann et al., 2013). The esiRNAs targeting either Luciferase (#RLUC) or P19 (#MU-220224-1) were purchased from Eupheria Biotech and used in combination with an empty vector expressing RFP (pDSV-RFP).

### Cell culture

HEK293 cells were kept in DMEM (Thermo Fisher, 31966-047) supplemented with 10% fetal bovine serum (Gibco, 10270106) and 1% penicillin/streptomycin. Cells were used between passages 5 and 15, and regularly checked for mycoplasma contamination. The day before transfection, 4 million cells were seeded in a 10 cm petri dish. For transfection, a 3:1 mix was prepared with polyethylenimine (Sigma, 408727) and DNA, and was added to the cells. After 24 h, the transfection mix was removed and cells washed twice with PBS and replenished with 10 ml of fresh medium without serum. After additional 24 h, ∼70% of the cells were transfected, and the conditioned medium and cell lysates collected. To discard detached cells and debris, the conditioned medium (10 ml) was centrifuged at 500 ***g*** for 10 min and supernatant collected (9 ml) and filtered with 0.22 µm filters at low speed (one drop per second). Filtered medium was concentrated using Amicon Ultra-15 Centrifugal Filter Unit (3 kDa cut-off; Millipore, UFC900308) at 5000 ***g*** for 1 h. About 300 µl of concentrated conditioned medium was recovered, aliquoted and stored at −80°C. Cells were lysed by scrapping in RIPA buffer supplemented with EDTA-free protease inhibitors (Roche, 04693159001). Lysed cells were centrifuged at maximal speed for 10 min, and supernatant collected, aliquoted and stored in the freezer until use. See below for the use of HEK293 cells on brain slices.

### Western blot

Protein lysates were denatured by heating at 70°C for 30 min with NuPAGE LDS sample buffer (Invitrogen, NP0007) and NuPAGE reducing agent (Invitrogen, NP0004). Samples were run in 4-12% Bis-Tris protein precast gels (Invitrogen, NP0335BOX) for 1 h and 15 min at a constant 165 V, using SDS running buffer MES (Invitrogen, NP0002). Proteins were transferred onto nitrocellulose membranes (0.45 µm pore size; Sigma, GE10600012) for 2.5 h at 400 mA constant, in MOPS buffer (Invitrogen, NP0001) with 15% methanol. As a reference for protein sizes, we used a pre-stained protein ladder (LI-COR, 928-60000). Immunodetection was carried out using the enhanced chemiluminescent method (Thermo Fisher, 34577).

### *In utero* electroporation

C57BL/6J wild-type (Janvier) mice were used, except for Fig. S1F, for which the *Btg2*::GFP line was used (Haubensak et al., 2004). The morning of the vaginal plug was defined as E0 and 13 days later (E13) mice were deeply anesthetized with isoflurane, their uterine horns exposed and ∼2 µl of plasmid (2 µg/µl with 0.01% of Fast Green) injected into the lateral ventricle of the embryonic brains. Electrodes were placed around the embryo head, with the anode facing the injection site, and six pulses of 30 V for 5 ms each were delivered using an electroporator (BTX ECM830). Afterwards, the uterus was relocated within the abdominal cavity, and the muscular walls and overlying skin were sutured independently. Mice were transferred to the housing box when fully awake and sacrificed at the indicated time points by cervical dislocation. Eventually, mice were administered with a single intraperitoneal dose of EdU (1 mg/kg) 24 h before sacrifice. All animal procedures were approved by local authorities and complied with all relevant ethical regulations (TVV 16/2018).

### Brain slice culture

Either naïve or *in utero* electroporated brains were dissected at E14 as described above and kept on ice-cold PBS with 0.5% penicillin/streptomycin (Gibco, 15140-122). The meninges were removed and brains immediately embedded in 4% low-melting point agarose (Carl Roth, 6351.2) and sliced using a vibratome (250 µm). Slices were transferred to culture inserts (Corning, 353090) pre-soaked in culture medium [Neurobasal; Thermo Fisher, 21103-049) supplemented with 10% horse serum (Thermo Fisher, 16050-130) and 1% penicillin/streptomycin]. For time-lapse imaging, we labelled microglia by adding isolectin B4 conjugated to an Alexa-488 fluorophore (Thermo Fisher, I21411) to the culture medium (5 µg/ml) 2 h before imaging started. A spinning disc microscope (Andor DragonFly) was used for live-imaging at 37°C and 5% CO_2_ with a time resolution of 15 min over a period of 12 h. For each slice, about 35 planes were acquired every 5 µm, and planes were later compiled into a maximal intensity projection for analysis by ImageJ (NIH) using the Manual Tracking plug-in. HEK293 cells for brain slices co-culture were transfected as indicated above and 2 days after transfection detached and resuspended in DMEM. Collagen type I-A (Wako, 631-00651) was freshly reconstituted and mixed with the cells to a final concentration of 1.5 mg/ml. Drops of cells embedded in collagen (50 µl) were seeded on parafilm and allowed to solidify for 30 min at 37°C. After solidification, the drops were placed adjacent to the pial side of naïve brain cortices and co-cultured for 24 h. For depletion of microglia, slices were incubated with liposomes (5 mg/ml) filled with PBS or clodronate (Liposoma, CP-005-005) for 48 h. At the indicated time points, the tissue was fixed with 4% PFA for 20 min and processed for immunohistochemistry.

### Immunohistochemistry, *in situ* hybridization and imaging

Embryo brains were dissected and fixed overnight by immersion in 4% paraformaldehyde (PFA) and vibratome sectioning (40 µm) obtained. Slices were permeabilized and blocked for 1 h at room temperature in blocking buffer (PBS 0.1% Triton X-100 and 5% donkey serum) and primary antibodies incubated for 3 nights at 4°C in blocking buffer with Alexa-conjugated secondary antibodies (Jackson ImmunoResearch) incubated overnight at 4°C in blocking buffer (Table S2). DAPI was used to counterstain nuclei and the Click-it reaction kit used to reveal EdU (Invitrogen, C10340). Immunohistochemistry was combined with *in situ* hybridization using the RNA-protein co-detection kit from RNAscope (323180) by following the manufacturer's instructions. P19 was detected using the Mm-4931414P19Rik-C1 probe, while the bacterial transcript dihydrodipicolinate reductase (DapB) was used as negative control (PN 320871). For quantification, single confocal planes were used. Tbr2 identified neuron progenitors, while apical (VZ) and basal (IZ, CP) Tbr2^–^ cells were scored as proliferating progenitors or neurons, respectively. To reliably associate gene expression with a given cell type, only discrete puncta within nuclei were scored. Images were acquired using an automated Zeiss ApoTome or confocal (LSM 780) microscope, and maximal intensity projections were quantified using ImageJ and Affinity Photo. For CD68 analysis, signal was obtained using the same settings for all the images (laser intensity, exposure time, gain, offset, etc.) and processed in ImageJ. To score CD68 mean gray intensity per microglia, the contour of Iba1^+^ cells were drawn using the polygon selection tool, each selection was then applied to the CD68 channel and the mean gray intensity measured. Next, a threshold (40 to 255) was applied to the CD68 signal, and the binary image was used to calculate the area covered by CD68 as a percentage of the Iba1^+^ area.

### Quantitative RT-PCR

Embryos electroporated *in utero* (E13) as before were dissected at E15, and the electroporated area dissected and dissociated into single cells, followed immediately by FACS (RPF^+^ DAPI^–^). RNA was obtained from these cells using the RNeasy micro kit (Qiagen, 74034) and reverse transcribed with the SuperScript IV VILO master mix (Thermo Fisher, 11756-050). The transcripts were quantified by qPCR using TB Green Premix Ex Taq (Takara, RR420L) on a QuantStudio 5 system (Thermo Fisher). Sequences of primers used were: P19 forward, 5′-TATGTGGCCTCTGAGGGTTC-3′; P19 reverse, 5′-TGTCTCTGAGGATGCCCTCT-3′. P19 expression levels were normalized to those of RFP using the following primers: forward, 5′-ATGAGGCTGAAGCTGAAGGA-3′; reverse, 5′-GTCCAGCTTGATGTCGGTCT-3′.

### Statistical analyses

Quantification of cell types and morphometric analyses were performed on at least three independent biological replicates and are depicted as mean±s.e.m. Statistical tests were performed using Prism9 (GraphPad). Details on statistical tests and significance are indicated in the figure legends.

## Supplementary Material

10.1242/develop.201574_sup1 Supplementary information
