# Supplementary material for: 4931414P19Rik, a microglia chemoattractant secreted by neural progenitors, modulates neuronal migration during corticogenesis
Source: Development. 2023 Apr 28;150(9):dev201574. doi: 10.1242/dev.201574 (PMC10163356; doi:10.1242/dev.201574)
Supplement: Supplementary information [file develop-150-201574-s1.pdf]

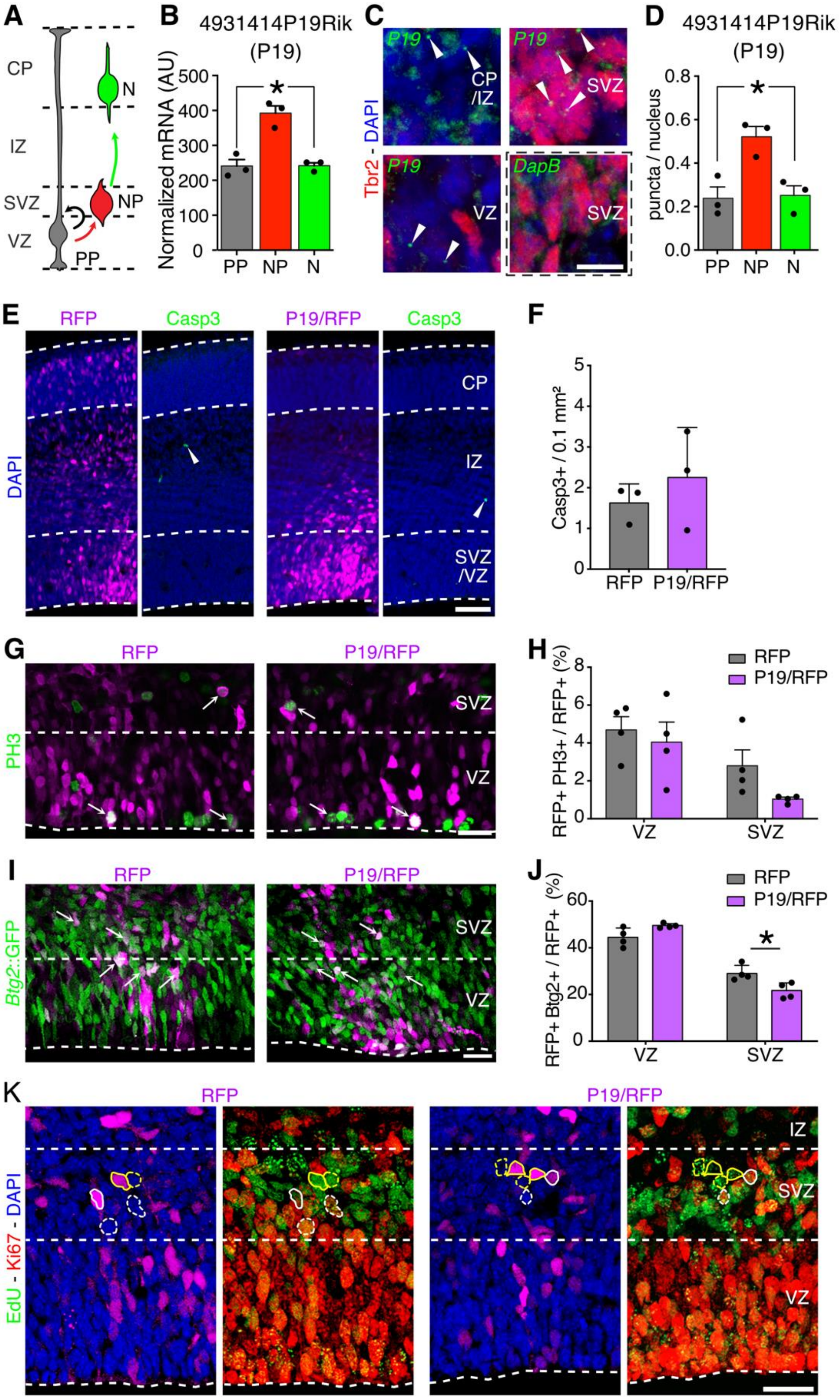

Development • Supplementary information

Fig. S1. The on-switch gene P19 does not affect neural survival or mitosis. (A and B) Drawing and quantification of P19 expression levels in proliferative and neurogenic progenitors and neurons (PP, DP and N, respectively) (from Aprea et al., 2013). (C) Combined fluorescent in situ hybridization with immunolabeling in E14 coronal brain sections to detect P19 mRNA (green) and the neuron progenitor marker Tbr2 (red). Arrowheads point to discreet P19 mRNA signal. The bacterial transcript DapB was used as a negative control. (D) Quantification of P19 mRNA per cell type. (E-J) Fluorescence pictures (E, G and I) and quantifications (F, H and J) of E15 brains two days after electroporation with control RFP or P19/RFP plasmids immunolabeled with markers of apoptosis (Casp3, arrowheads), mitosis (PH3), or a reporter gene marker of neurogenic commitment (*Btg2*::GFP) as indicated. Arrows point to double positive cells. (K) Low magnification panels of those shown in Fig. 1E. Quantifications are depicted as bar graphs with individual values ± SEM. Either a Benjamini–Hochberg test (B), a one-way ANOVA and Tukey's post-hoc test (D), a two-tailed unpaired t test (F), or a two-way ANOVA and Bonferroni's post-hoc test (H, J) were used to assess significance (* $p < 0.05$). Scale bars = 10 μm (C), 50 μm (E), 25 μm (G, I, K).

Development • Supplementary information

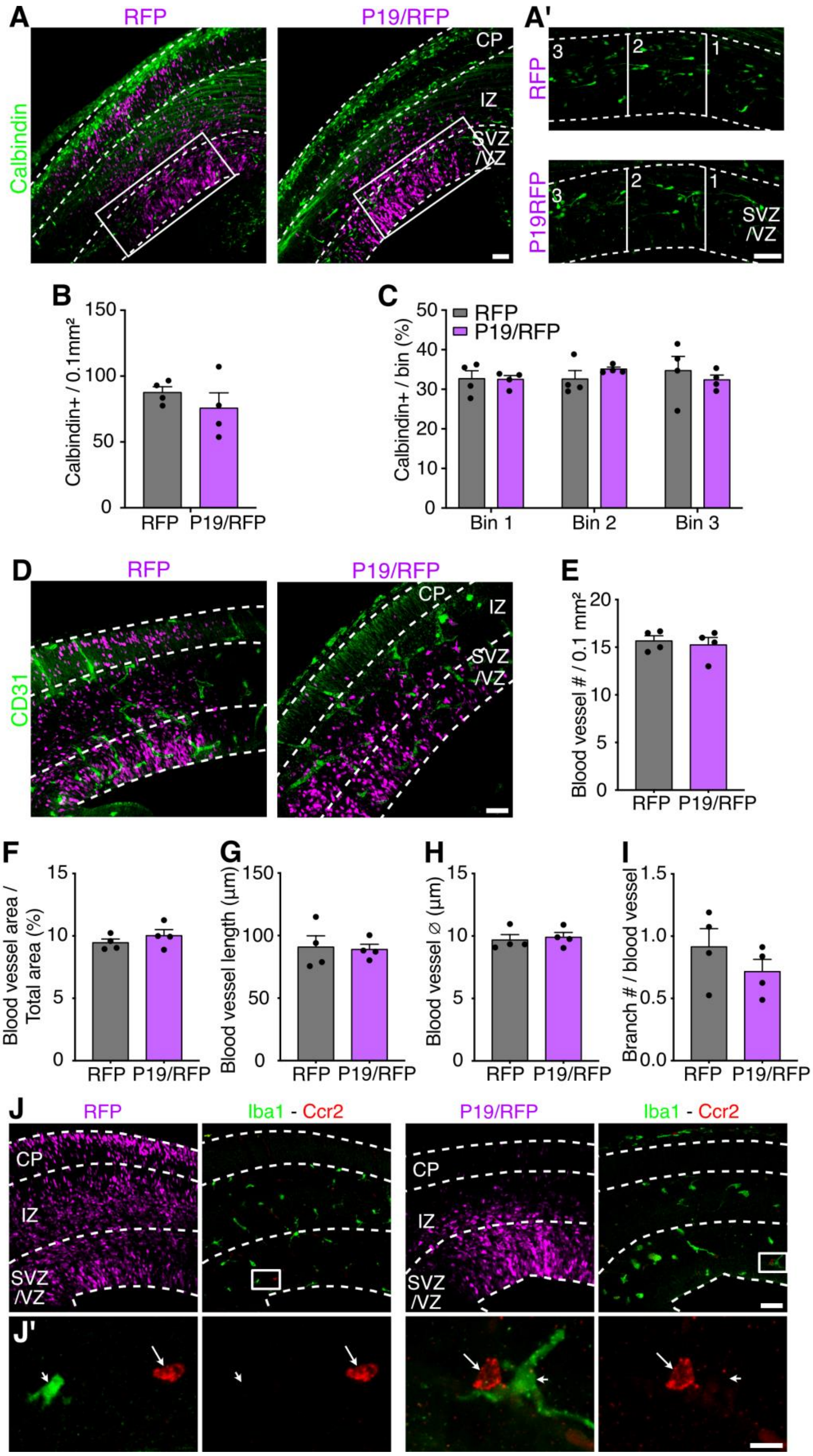

Development • Supplementary information

Fig. S2. P19 overexpression does not affect interneuron migration nor angiogenesis. (A and D) Fluorescence images and corresponding quantifications (B, C, E-I) of coronal sections of E15 brains two days after electroporation with control or P19/RFP plasmids and immunolabeled with Calbindin or CD31 (as indicated) to assess interneuron migration and blood vessels architecture, respectively. Insets (A) are magnified in (A') and continuous lines delimit bins perpendicular to the ventricular surface (A'). (J) Fluorescence images of electroporated brain sections counterstained with Iba1 (green, arrowheads) and the infiltrating macrophage marker Ccr2 (red, arrows). Quantifications are depicted as bar graphs with individual values ± SEM. Either a two-way ANOVA and Bonferroni's post-hoc test (C), or a two-tailed unpaired t test (B, E-I) were used to assess significance. Scale bars = 50 μm (A, A', D, J), 10 μm (J').

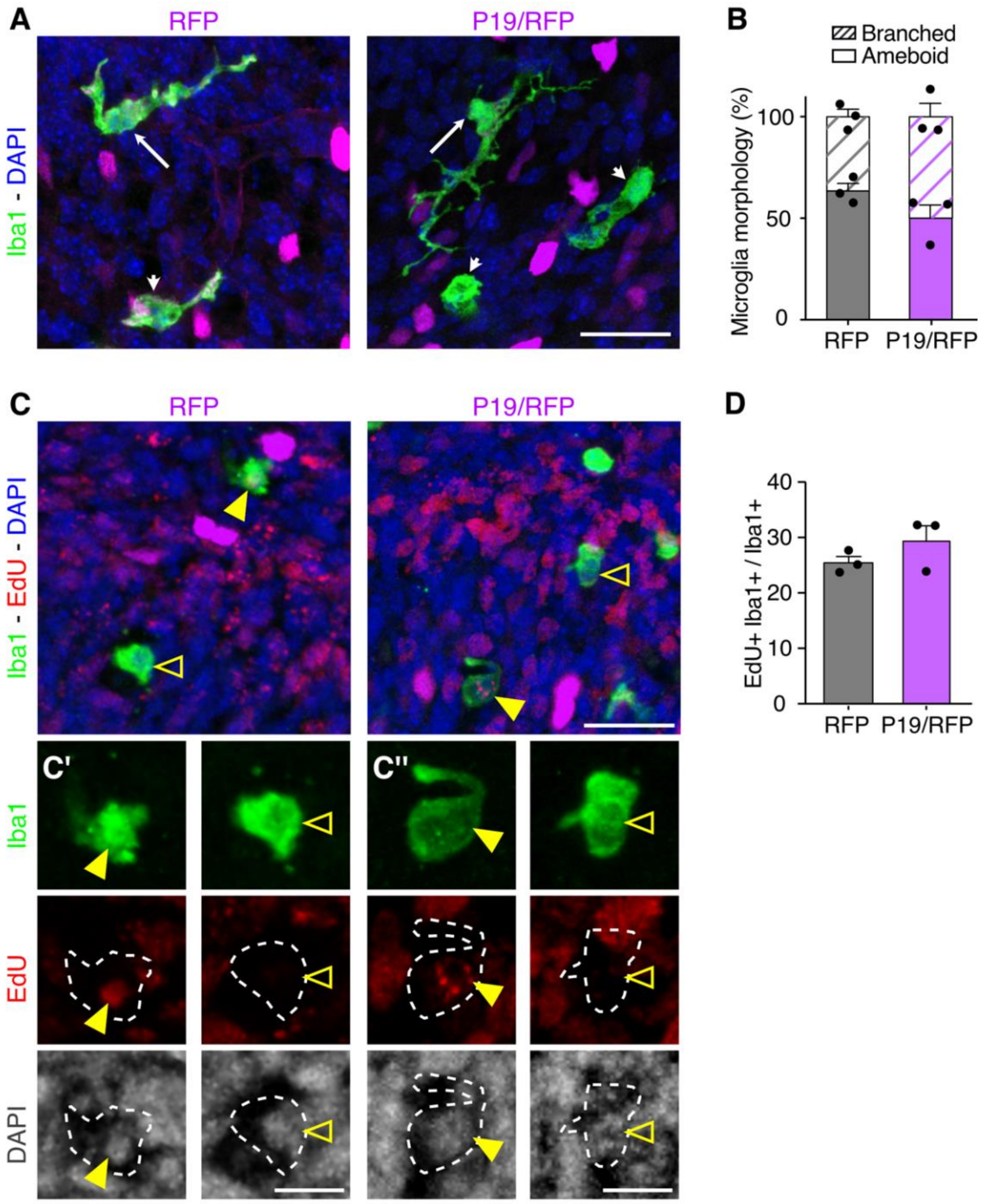

Fig. S3. P19 overexpression does not affect microglia morphology nor proliferation. (A and C) Fluorescence images and corresponding quantifications (B, and D) of coronal sections of E15 brains two days after electroporation with control RFP or P19/RFP plasmids and immunolabeled with Iba1 and EdU (as indicated) to assess microglia morphology or proliferation, respectively. Single-channel higher magnifications are shown in (C' and C''). Branched or amoeboid microglia are pointed by white arrows or arrowheads, respectively. Iba1+ and EdU+ cells are pointed with a yellow arrowhead, while Iba1+ and EdU– cells are indicated with an empty arrowhead. Quantifications are depicted as bar graphs with individual values ± SEM. A two-tailed unpaired t test was used to assess significance. Scale bars = 25 μm (A, C), 10 μm (C', C'').

Development • Supplementary information

Table S1. Predicted features of P19 amino acid sequence. Scores are given together with the maximal score or the cut-off value into parenthesis. NLS, nuclear localizing signal; SP, signal peptide; CS, cleavage site.

Feature	Position	Sequence	Score	Resource
NLS	291-327	RGTGQKNSRRKRDLVLSKLVHNVHNHITNDKRFNGS	4.4 (max 10)	NLS Mapper
NLS	369-400	FLTKRREYRNSLNPFKGLKEKEEKKLRSRRY	4.6 (max 10)	NLS Mapper
NLS	295-302	QKNSRRKR	> 0.3 (max 1)	NLStradamus
NLS	372-402	KRREYRNSLNPFKGLKEKEEKKLRSRRYRLF	> 0.3 (max 1)	NLStradamus
SP	1-17	MSFSATILFSPPSGSEA	79 (max 100)	TOPCONS
SP	1-17	MSFSATILFSPPSGSEA	0.99 (max 1)	Phobius
CS	17-18	AR	0.62 (cut-off 0.5)	PrediSi
CS	17-18	AR	0.52 (cut-off 0.5)	SignalP

Table S2. List of primary antibodies used in this study.

Antibody	Host	Manufacturer	Cat. #	RRID	Dilution
Calbindin	rabbit	Swant	CB-38	AB_10000340	1:1000
Caspase3	rabbit	BD Biosciences	559565	AB_397274	1:600
Ccr2	rabbit	Abcam	ab273050	AB_2893307	1:200
CD206	rat	Biolegend	141708	AB_10900231	1:200
CD31	rabbit	Abcam	ab222783	AB_2905525	1:500
CD68	mouse	Abcam	ab955	AB_307338	1:500
Ctip2	rat	Abcam	ab18465	AB_2064130	1:600
Flag	mouse	Sigma	F1804	AB_262044	1:1000
GAPDH	mouse	Novus Biologicals	NB300221	AB_10077627	1:1000
Iba1	rabbit	WAKO	019-19741	AB_839504	1:600
Iba1	goat	WAKO	011-27991	N/A	1:400
Ki67	rabbit	Abcam	ab833	AB_306483	1:500
Lyve1	rat	Invitrogen	13-0443-82	AB_1724157	1:200
P2ry12	rabbit	Abcam	ab300141	N/A	1:400
PH3	rat	Abcam	ab10543	AB_2295065	1:600
RFP	rabbit	Rockland	600-401-379	AB_2209751	1:600
RFP	rat	Chromotek	5F8	AB_2336064	1:600
Tbr2	rabbit	Abcam	ab23345	AB_778267	1:500

Secondary antibodies were purchased from Jackson ImmunoResearch and diluted 1:500 (catalogue numbers: 712-545-150, 705-545-003, 715-545-151, 712-165-150, 711-165-152, 715-165-151, 712-605-153, 711-605-152, 715-605-151).
