# Supplementary material for: 4931414P19Rik, a microglia chemoattractant secreted by neural progenitors, modulates neuronal migration during corticogenesis
Source: Development. 2023 Apr 28;150(9):dev201574. doi: 10.1242/dev.201574 (PMC10163356; doi:10.1242/dev.201574)
Supplement: Supplementary file 1 [file develop-150-201574_review_history.pdf]

4931414P19Rik, a microglia chemoattractant secreted by neural progenitors, modulates neuronal migration during corticogenesis

Ivan Mestres and Federico Calegari
DOI: 10.1242/dev.201574

Editor: Debra Silver

Review timeline

Submission to Review Commons:	23 May 2022
Submission to Development:	22 December 2022
Editorial decision:	30 January 2023
First revision received:	24 February 2023
Editorial decision:	16 March 2023
Second revision received:	27 March 2023
Accepted:	2 April 2023

Reviewer 1

Evidence, reproducibility and clarity

This study by Mestres et al. made the first attempt to investigate the role of P19, an upregulated transcript in neuronal progenitors during the corticogenesis discovered by the same group, in regulating cortical neurogenesis and microglia behaviour between E13-15 in mouse embryos. They used in-utero electroporation to overexpress P19 in progenitors residing in VZ/SVZ and showed that up-regulation of P19 could inhibit the migration of new born cortical neurons into the CP. Furthermore, they found that P19 may act as a chemoattractant to microglia. Their in vitro experiments further suggests that P19 is a secreted protein that can attract microglia which contributes to the migration of neuronal progenitors and/or newly generated neurons to CP. This work added knowledge to P19 functions which were largely unknown and also provide new evidence of microglial regulating corticogenesis.

Major concerns:

1. The authors employed an overexpression of P19 system by in-utero electroporation of P19-expression plasmids. However, the expression levels of P19 in both groups (RFP and P19) were not shown. Therefore, it is hard to link the current phenotype to P19 expression in a more qualitative manner. qPCR or ISH results will be required. In line with this point, the expression level of P19 in the HEK cells used in the co-culture model is not known, either.

2. As the authors indicated, P19 is already upregulated in neuronal progenitors during the examined embryonic period. In the current work, the authors even increased the expression of P19 on top of the originally upregulated level. On the other hand the authors suggest that P19 is a secreted factor. It happens quite often to observe divers or even paradoxical effects of cytokines at high or low expression levels (concentrations). Therefore, loss-of-function experiments (e.g. siRNA knockdown) would be essential to confirm current results.

3. If overexpression of P19 could cell-extrinsically inhibit the migration of newborn neurons, a reduced size of CP would be expected. However, from the images (e.g. Fig. 1A), the size of CPs seem to be similar in both groups. In addition, deep layer makers (e.g. Tbr1, Ctip2) should investigated.

© 2023. Published by The Company of Biologists under the terms of the Creative Commons Attribution License (https://creativecommons.org/licenses/by/4.0/).

4. In Fig. 2D, E, microglia density of P19 group is higher in VZ/SVZ. However, the proliferation was not altered. The authors concluded that this was due to the migration of microglia towards the VZ/SVZ. Therefore, a reduced density of microglia in IZ is expected, but the authors showed that no change in IZ (seems even a tendency to increase). How to explain that? Moreover, did the authors observe migration of microglia from non-transfected area to transfected area in their time-laps imaging? In parallel, did they observe the reduced migration of RFP+ cells in the P19 group from the time-laps imaging data?

5. In the co-culture experiment, I understood it as the authors wanted to suggest the diffusion of P19 from pial surface. However, it is also possible that P19 in HEK cells can be released in to the medium to have trigger cells in the brain slices. If there is no available antibodies against P19 in the moment, it would be nice to use their Flag-tagged P19 in this experiment and show the gradient patter of immunostaining of Flag-P19 in the slices. In addition, there seemed to be even more Iba1+ cells in the CP of RFP group than P19 group (Fig. 3B).

6. For the co-culture experiment, RFP+Clodronate control is missing.

Minor concerns:

1. L163. "our analyses revealed that the physiological expression of the up-switch". In the current work, overexpression of P19 was used, thereby it was not physiological.
2. The promoter information of the P19 plasmids should be provided.
3. The title of the manuscript does not reflect the whole work.
4. The two groups are named RFP and P19, but actually in P19 group the transfected cells are also RFP. This can cause some confusion when read data in e.g. Fig. 1B, D.

Referees cross-commenting

I am happy to see that my and Reviewer 2's comments partially overlap. I also agree with other suggestions from Reviewer 2.

Significance

Although more detailed mechanisms are not yet clear, this work is the first functional analysis of P19 during brain development, suggesting that neuronal progenitor cells, in terms of secreting proteins, are also regulating microglia population which in turn affects the corticogenesis. This is also in line with previous work showing microglia are involved in sculpturing neural network. This work would be interesting to scientists working on brain development.
My expertise: glia development and function.

Reviewer 2

Evidence, reproducibility and clarity

Summary:

Mestres and Calegari use C57BL/6J mice to demonstrate that the uncharacterized transcript 4931414P19Rik (P19 for short) is up-regulated by neurogenic progenitors during mouse corticogenesis. By over-expressing P19 during embryogenesis in the cortex using in utero electroporation, the authors demonstrate that P19 over-expression results in the accumulation of microglia near P19-expressing cells and this ultimately inhibits neuronal migration.

Major Comments:

1. While the authors present P19 expression levels in proliferative and neurogenic progenitors and neurons from their previously published findings in Summplemental Figure 1A-B, it is important that we visualize the expression pattern of P19 across the cortex (VZ/SVZ, IZ, CP). Therefore, the authors need to use something like RNAScope (e.g., ACD--where the company can design in situ probes for you) to show P19 expression in both high and low magnification images across the cortex and use unique markers for each of these cell types to demonstrate that P19 is indeed uniquely

© 2023. Published by The Company of Biologists under the terms of the Creative Commons Attribution License (https://creativecommons.org/licenses/by/4.0/).

highly expressed in neurogenic progenitors. By throwing on a probe for microglia, the authors can also show if microglia somas are in close-proximity to P19 expressing cells in the cortex under normal (non-IUE) conditions.

2. If the authors are observing increased Iba1+ microglia in the VZ/SVZ but not a loss of microglia in the IZ, where are they coming from and are these indeed microglia or infiltrating macrophages? To address these questions, the authors need to evaluate microglia numbers across various regions of the cortex to see if these cells are lost somewhere else at the expense of this accumulation. Also, the authors need to use microglia specific markers (e.g., P2ry12, Fcrls) to show that these are indeed microglia, as Iba1 also marks macrophages. Similarly, the authors should use some macrophage markers (e.g., Ccr2, etc.) to show that these are not macrophages that have infiltrated the cortex. The authors should also look at Rosin and Kurrasch 2018 Journal of Neuroinflammation (impact of IUE on hypothalamic microglia) to see if IUE itself in the cortex is having any impact on microglia.

3. To understand the importance of these phenotypes (e.g., microglia accumulation & disrupted neuronal migration), the authors need to look beyond 48 hours after IUE (e.g., look at E17 & birth) to see if this is long lasting or transient. This will demonstrate the significance of the findings (i.e., transient--more of a subtle impact perhaps on connection vs. long-lasting--a prominent impact on patterning), especially with claims in the discussion about neurodevelopmental disorders and human mutations in P19 being linked to schizophrenia.

4. The authors need to pick a better representative image for Figure 3B as it does not match what is quantified. It looks like there is a large number of microglia in the CP of RFP controls, which is much greater than what is seen in P19 (i.e., would go against the argument that P19 over-expression from HEK cells is attracting microglia if the regular RFP HEK cells are doing this). On a similar note, in control sections in Figure 2 it looks like there are almost no microglia in the CP and now in these slice experiments there appears to be a number of microglia in the area. It is important that the authors quantify CP microglia numbers in their slices for Figure 2 and compare this to what they are getting in Figure 3 to see if the experimental method (e.g., slice culture/ex vivo preparation) itself is making microglia accumulate in the CP.

Minor Comments:

1. For almost all of the figures (e.g., Figure 1C, Supplemental Figure 1) the authors need different coloured arrows pointing to single vs. double positive cells (there are a lack of arrows pointing to what should be focused on in the Figures).
2. Figure 1E, please include a lower magnification image so we can observe an appropriate area for comparison to the quantification. Please also include DAPI in these images so we can see the nuclei of the individual cells.
3. For the analysis of microglia morphology in Supplemental Figure 2J, please include example images of what was considered amoeboid and what was considered ramified, in addition to including high magnification images from control and P19 over-expression animals.
4. The authors need to show both control and P19 over-expression IUE images for Supplemental Figure 2K.
5. In the text (line 292) the authors refer to Figure 2M but it should be 2J.
6. In the text (e.g., discussion) the authors should also include publications that discuss microglia-neural progenitor interactions outside of the cortex, as this is not a unique to the cortex (e.g., Rosin et al., 2021 Developmental Cell - microglia-progenitor interactions in the hypothalamus).

Referees cross-commenting

It looks like some of the comments that I made overlap with comments that were made by Reviewer 1, which is nice to see and demonstrates that there are key areas that need to be addressed by revising the current study/manuscript, including additional experimentation. I also agree with the other suggestions made by Reviewer 1, and together agree that the authors will likely need 3-6 months to revise the study/manuscript.

© 2023. Published by The Company of Biologists under the terms of the Creative Commons Attribution License (https://creativecommons.org/licenses/by/4.0/).

Significance

The findings that Mestres and Calegari present in this manuscript are important to the microglia field as work on microglial roles during neurodevelopment (e.g., embryogenesis) are lacking--especially as it relates to how they interact with neighbouring cells to impact neurodevelopmental programs. Moreover, the authors use some very interesting and unique methodologies (e.g., live-cell imaging, ex vivo culture with cells, etc.) that will be helpful in advancing the microglia field for research conducted in the embryonic cortex.

Reviewer expertise: Microglial functions during embryogenesis (methods: IHC/ISH, IUE, live-cell imaging, ex vivo slice culture, etc).

Author response to reviewers' comments

POINT-BY-POINT RESPONSE TO THE REVIEWERS

Authors' general statement

We much appreciate the reviewers' dedication and time in evaluating our manuscript. Their concerns were very constructive and helped us to substantially improve the quality of the work and interpretation of the results. Below, Reviewers will find a point-by-point reply to their comments including the addition of several new experiments accompanied by an entirely revised manuscript including new experiments and figures. We believe that this led to a substantially improved manuscript within which all changes were highlighted in red. We hope that this revised version will be to the reviewers' satisfaction and leading to a timely dissemination of our findings.

REVIEWER 1
This study by Mestres et al. made the first attempt to investigate the role of P19, an upregulated transcript in neuronal progenitors during the corticogenesis discovered by the same group, in regulating cortical neurogenesis and microglia behaviour between E13-15 in mouse embryos. They used in-utero electroporation to overexpress P19 in progenitors residing in VZ/SVZ and showed that up-regulation of P19 could inhibit the migration of new born cortical neurons into the CP. Furthermore, they found that P19 may act as a chemoattractant to microglia. Their in vitro experiments further suggests that P19 is a secreted protein that can attract microglia which contributes to the migration of neuronal progenitors and/or newly generated neurons to CP. This work added knowledge to P19 functions which were largely unknown and also provide new evidence of microglial regulating corticogenesis.

Major concerns:

Reviewer 1, Point 1.

The authors employed an overexpression of P19 system by in-utero electroporation of P19-expression plasmids. However, the expression levels of P19 in both groups (RFP and P19) were not shown. Therefore, it is hard to link the current phenotype to P19 expression in a more qualitative manner. qPCR or ISH results will be required. In line with this point, the expression level of P19 in the HEK cells used in the co-culture model is not known, either.

Authors reply
We thank the reviewer for this comment, which we address together with a very similar one from reviewer 2 (major comment 1). By capitalizing on tools publicly available (NCBI and proteinatlas.org), as it can be seen in the figure for the reviewers (R) below (Fig. R1; top), HEK293 is among the cell lines expressing the lowest levels of P19 implying that our use of plasmids with a strong constitutive promoter would trigger its up-regulation by orders of magnitude. A similar picture arises in comparing P19 expression in various organs, including the brain (Fig. R1; bottom). In this case, however, the reviewer should consider that a more meaningful comparison should assess different cell types of any given organ, rather than the

© 2023. Published by The Company of Biologists under the terms of the Creative Commons Attribution License (https://creativecommons.org/licenses/by/4.0/).

whole organ. This is indeed shown for different cell types of the developing mouse brain in the revised Fig. S1A-D which now also includes the additional validation requested by fluorescent ISH together with immunohistochemistry for the basal progenitor marker Tbr2. Finally, we now also report on P19 expression in the developing human brain by independently published work providing single-cell transcriptome analysis (line 113; Cao et al., 2019; Telley et al., 2019). As the reviewer can see, all approaches and published resources are fully consistent, both at the qualitative and quantitative level, with our original claim derived from our double RFP/GFP reporter mouse line (Aprea et al., 2013).

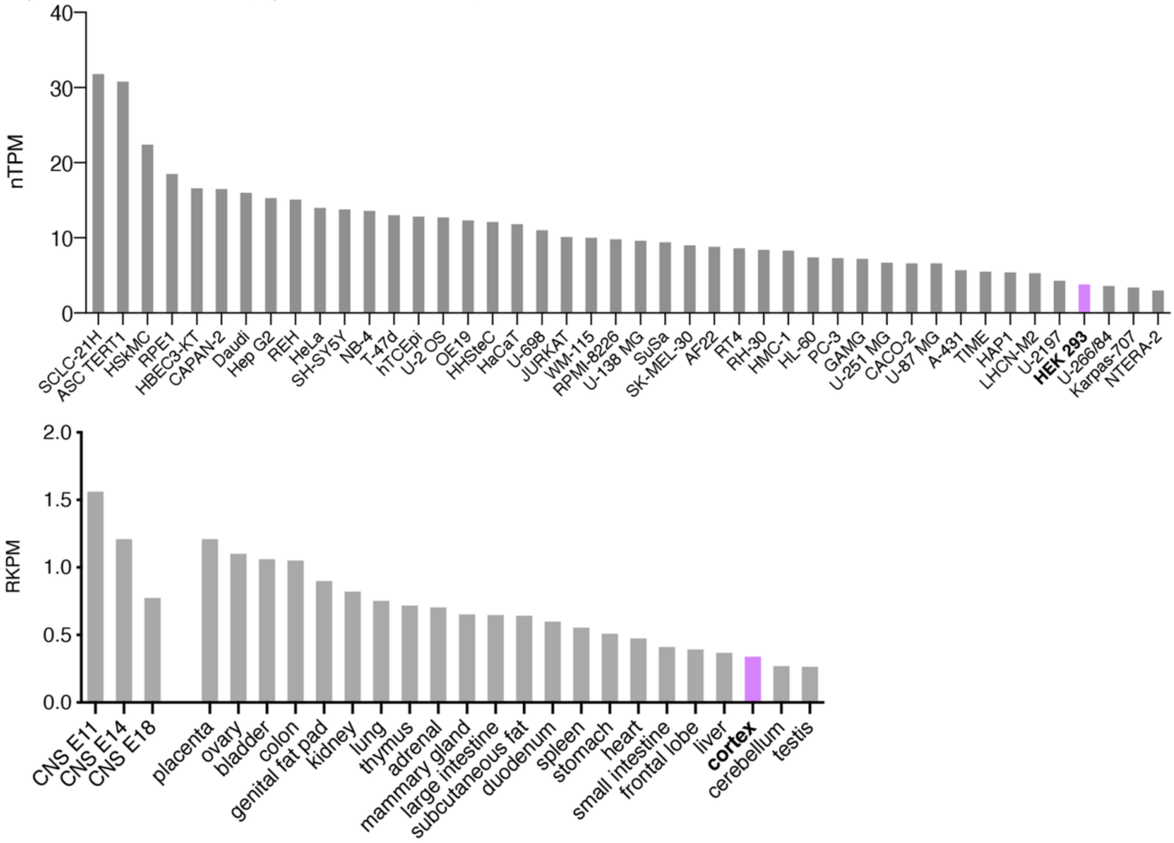

***Figure R1** Graphs representing P19 expression across several human cell lines including HEK293 (top) and mouse organs (bottom). Data taken from Uhlen et al., 2015 and Yue et al., 2014.*

Reviewer 1, Point 2.
As the authors indicated, P19 is already upregulated in neuronal progenitors during the examined embryonic period. In the current work, the authors even increased the expression of P19 on top of the originally upregulated level. On the other hand the authors suggest that P19 is a secreted factor. It happens quite often to observe divers or even paradoxical effects of cytokines at high or low expression levels (concentrations). Therefore, loss-of-function experiments (e.g. siRNA knockdown) would be essential to confirm current results.

Authors reply
The reviewer is raising an important point on the value to perform converse experiments with P19 knock-down. Before addressing this, we would like to elaborate a little more on the notion that P19 may be expressed at *"unphysiologically high levels"* resulting in unspecific effects. While this possibility always exists in all studies based on gene overexpression, it should be noted that P19 in physiological conditions is expressed at *"high levels"* only by basal progenitors in the SVZ. Conversely, both apical progenitors in the VZ and newborn neurons in the IZ/CP express P19 at *"low levels"* (Supp. Fig. 1A-D). As a result, our overexpression strategy by electroporation, which almost exclusively targets apical progenitors in the VZ, would ultimately raise P19 levels primarily within cells that otherwise express low P19. While potentially still at *"unphysiologically high levels"* within this targeted pool of cells, only about 20-30% of cells within the VZ are targeted by electroporation resulting in a *"dilution effect"* of P19 protein secreted within this layer due to the presence of 70-80% of cells that are not targeted. In other

© 2023. Published by The Company of Biologists under the terms of the Creative Commons Attribution License (https://creativecommons.org/licenses/by/4.0/).

words, we believe that the primary effect of our approach is to miss-localize P19 protein within the VZ at levels perhaps similar to that of the SVZ in physiological conditions. We would love to be able to directly assess this but, unfortunately, quantification of extracellular P19 in specific cortical layers by quantitative mass-spec or other methods is currently not possible.

Back to knock-down experiments: To address this, we attempted to knock-down P19 in vivo by using two alternative approaches to RNAi. First, we performed in utero electroporation with a shRNA plasmid targeting P19 and previously validated in HEK293 cells to result in a 70% silencing efficiency (not shown). While technically effective, this approach did not result in any difference in terms of Iba1+ cells within the targeted area as compared to electroporation with a control shRNA plasmid (Luc) or other control conditions (e.g. naïve brains, electroporation with empty vectors, etc.). We reasoned, however, that this negative result was inconclusive for the simple reason, as mentioned above, that apical progenitors already express low levels of P19 and that electroporation only targets about 20-30% of cells. Given that P19 effects are cell-extrinsic, its levels already low in the VZ, and electroporation reducing it further only within a small fraction of cells, it is little surprise that RNAi was insufficient to trigger any observable effect.

Hence, second, we attempted another RNAi approach by electroporation with enzymatically-prepared small-interfering RNAs (esiRNAs) as a cocktail of digested fragments rather than encoded within a plasmid expression vector. In fact, this approach was originally reported to trigger RNAi homogeneously within tissues, as opposed to mosaic targeting within 20-30% of cells, by virtue of a greater diffusion of smaller esiRNA molecules compared to much larger plasmids (Calegari, et al., 2002). To our surprise, this esiRNA approach triggered an increased density of Iba+ cells not only when targeting P19 but also when using control esiRNA against luciferase (Fig. R2). We are puzzled by this outcome suggesting that esiRNAs unspecific/off-target effects invariably activate microglia irrespective on the cocktails used. This may not be entirely surprising if small esiRNAs were to diffuse within the extracellular space of the VZ, which is not the case when using plasmid DNA. The nature of this unspecific effect, and whether or not it is limited to microglia remains unclear, but we found it important to report it in our revised manuscript as a warning to the community using this method (page 289).
Remains the fact, that neither of the two RNAi approaches resulted in any conclusive result. In the future, effects of P19 depletion would need to be investigated by more laborious methods, such as KO mice, not feasible within the current study.

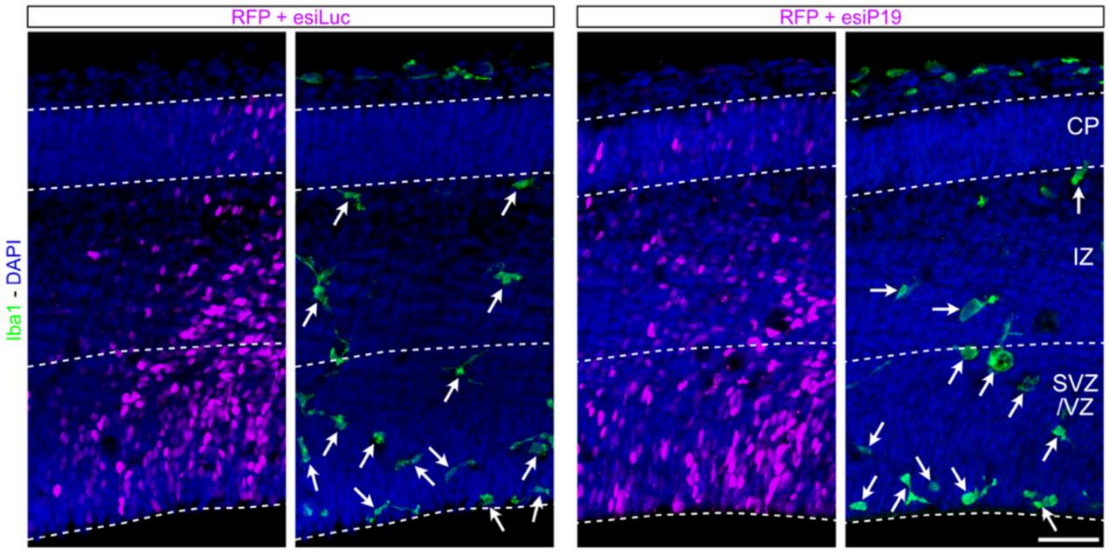

Figure R2. *Co-electroporation of RFP (magenta) together with esiRNAs against either luciferase or P19. Note the higher density of Iba1+ cells (green) in both conditions relative to control brains (e.g. Fig. S2J). Scale bar 50 μm.*

© 2023. Published by The Company of Biologists under the terms of the Creative Commons Attribution License (https://creativecommons.org/licenses/by/4.0/).

Reviewer 1, Point 3.
If overexpression of P19 could cell-extrinsically inhibit the migration of newborn neurons, a reduced size of CP would be expected. However, from the images (e.g. Fig. 1A), the size of CPs seem to be similar in both groups. In addition, deep layer makers (e.g. Tbr1, Ctip2) should investigated.

Authors reply
We agree with the reviewer that a thinner CP is expected as a result of a slower neuronal migration. However, soon after electroporation at E15 this effect may be too small to be appreciated. To address this, we analyzed brains at E18 and, taking on the good suggestion by this reviewer and reviewer 2 (point 3), combined the analysis with staining for layer markers (Ctip2). Indeed, this highlighted subtle changes in cortical layers thickness upon P19 overexpression and, particularly, a thinner layer VI mainly constituted by neurons generated after electroporation. These new data were added as new Fig. 5D of the revised manuscript and Fig. R3 below).

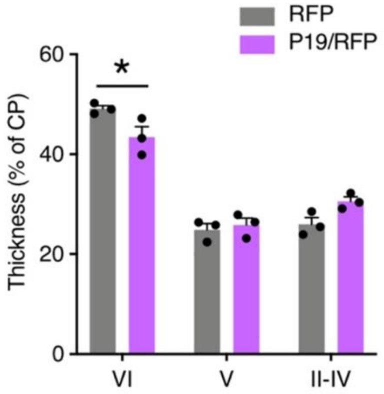

***Figure R3**. Thickness of cortical layers as a percentage of total cortical thickness. * p= 0.03 ANOVA and Bonferroni post-hoc test.*

Reviewer 1, Point 4.
In Fig. 2D, E, microglia density of P19 group is higher in VZ/SVZ. However, proliferation was not altered. The authors concluded that this was due to the migration of microglia towards the VZ/SVZ. Therefore, a reduced density of microglia in IZ is expected, but the authors showed that no change in IZ (seems even a tendency to increase). How to explain that? Moreover, did the authors observe migration of microglia from non-transfected area to transfected area in their time-laps imaging? In parallel, did they observe the reduced migration of RFP+ cells in the P19 group from the time-laps imaging data?

Authors reply
Certainly, we agree that given the comparable proliferation of Iba1+ cells, their accumulation must result in a decreased density somewhere else and/or infiltration of macrophages from the blood vessels and/or activation of other non-parenchymal macrophage populations (concerning this important distinction between microglia versus macrophages, please refer to the comment and our reply to Reviewer 2, point 2). Our time-lapse microscopy experiments indeed showed that microglia migrate at a faster pace within the transfected area. In addition, new quantifications of transfected versus untransfected neighboring areas of the same brain included in our revised manuscript show that an increase in Iba1+ cell in the former is mirrored by a converse decrease in the latter with both the ventral and dorsal regions adjacent to the electroporated area containing a reduced density of Iba1+ cells relative to control brains (Fig. 3 G and H). Regarding migration of RFP+ cells, our time-lapse imaging was performed within a field of the proliferative areas VZ/SVZ, where most of the Iba1+ cells reside, and not in the IZ where new-born neurons undergo radial migration toward the CP.

Reviewer 1, Point 5.
In the co-culture experiment, I understood it as the authors wanted to suggest the diffusion of P19 from pial surface. However, it is also possible that P19 in HEK cells can be released in to the medium to have trigger cells in the brain slices. If there is no available antibodies against P19

© 2023. Published by The Company of Biologists under the terms of the Creative Commons Attribution License (https://creativecommons.org/licenses/by/4.0/).

in the moment, it would be nice to use their Flag-tagged P19 in this experiment and show the gradient patter of immunostaining of Flag-P19 in the slices. In addition, there seemed to be even more Iba1+ cells in the CP of RFP group than P19 group (Fig. 3B).

Authors reply
The reviewer is correct, no antibody against P19 is currently available. We attempted what the reviewer suggested and performed anti-Flag immunolabeling in the experiment described but no P19 gradient was discernible even after extensive troubleshooting including testing different anti-Flag antibodies (Sigma clone M2 and Santa Cruz clone H- 5) and different antigen retrieval methods (0.1M citrate buffer, 1h at 70°C; 0.1 mM EDTA 10 min at 37°C; and DAKO retrieval solution 20 min at 99°C). Our inability to detect a clear P19 gradient, actually two, is not surprising as a quantitative read out of protein concentration in tissue resulting from secretion of P19 both from i) HEK293 cells at the pial surface and ii) basal progenitors within the SVZ seems particularly challenging. Independently from this, it should be noted that in a physiological context in which P19 is primarily secreted by basal progenitors, due to the fact that basal progenitors are the cells expressing the highest levels of P19 (Fig. S1A-D), our conclusion that P19 forms one gradient appears to be necessarily correct since no other source of P19 would be present.

Correct, the example shown in the previous version of the manuscript had the highest Iba1+ cell density within the CP (see red arrow pointing to the extreme value in the distribution in Fig. R4; right). We changed the panel for a more representative one and thank the reviewer for pointing this out.

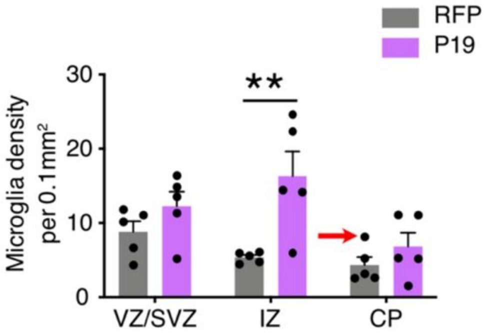

***Figure R4** Counts of individual samples including reference to former panel 3B (red arrow).*

Reviewer 1, Point 6.
For the co-culture experiment, RFP+ clodronate control is missing.

Authors reply
The RFP + clodronate control has now been added (Fig. 4 D and E).

Reviewer 1, minor concerns
L163. "our analyses revealed that the physiological expression of the up-switch". In the current work, overexpression of P19 was used, thereby it was not physiological.
The sentence refers to Fig. S1, in which we describe P19 as a physiological up-switch gene.

The promoter information of the P19 plasmids should be provided.

The expression of both, RFP and P19 were under independent simian virus 40 (SV40) promoters. Information has now been added in the Methods section. Line 524.

The title of the manuscript does not reflect the whole work.
This is true, the title was changed

The two groups are named RFP and P19, but actually in P19 group the transfected cells are also RFP. This can cause some confusion when read data in e.g. Fig. 1B, D.
Now, both in the text and images, it reads RFP and P19/RFP to reflect that there is RFP expression under both conditions.

© 2023. Published by The Company of Biologists under the terms of the Creative Commons Attribution License (https://creativecommons.org/licenses/by/4.0/).

CROSS-CONSULTATION COMMENTS
I am happy to see that my and Reviewer 2's comments partially overlap. I also agree with other suggestions from Reviewer 2.

Reviewer #1 (Significance)
Although more detailed mechanisms are not yet clear, this work is the first functional analysis of P19 during brain development, suggesting that neuronal progenitor cells, in terms of secreting proteins, are also regulating microglia population which in turn affects the corticogenesis. This is also in line with previous work showing microglia are involved in sculpturing neural network. This work would be interesting to scientists working on brain development.

Reviewer #2
Mestres and Calegari use C57BL/6J mice to demonstrate that the uncharacterized transcript 4931414P19Rik (P19 for short) is up-regulated by neurogenic progenitors during mouse corticogenesis. By over-expressing P19 during embryogenesis in the cortex using in utero electroporation, the authors demonstrate that P19 over-expression results in the accumulation of microglia near P19-expressing cells and this ultimately inhibits neuronal migration.

Major Comments:
Reviewer 2, Point 1.
While the authors present P19 expression levels in proliferative and neurogenic progenitors and neurons from their previously published findings in Supplemental Figure 1A-B, it is important that we visualize the expression pattern of P19 across the cortex (VZ/SVZ, IZ, CP). Therefore, the authors need to use something like RNAScope (e.g., ACD--where the company can design in situ probes for you) to show P19 expression in both high and low magnification images across the cortex and use unique markers for each of these cell types to demonstrate that P19 is indeed uniquely highly expressed in neurogenic progenitors. By throwing on a probe for microglia, the authors can also show if microglia somas are in close-proximity to P19 expressing cells in the cortex under normal (non-IUE) conditions.

Authors reply
This point is related to reviewer 1, comment 1. Following the Reviewers' suggestions, we performed fluorescence ISH to reveal P19 in combination with immunolabeling either for the macrophage marker Iba1 or basal progenitor marker Tbr2 (the latter is now included in the revised Fig. S1C and D). Below, the reviewer can see low magnifications of the panels shown in Fig. S1C. Note that blood vessels and the cortical plate/meninges were highly reactive to the RNAScope in situ probes (either P19 or negative control, DapB) resulting in strong autofluorescence (Fig. R4; green). For this reason, only high magnification images were chosen for the main manuscript.

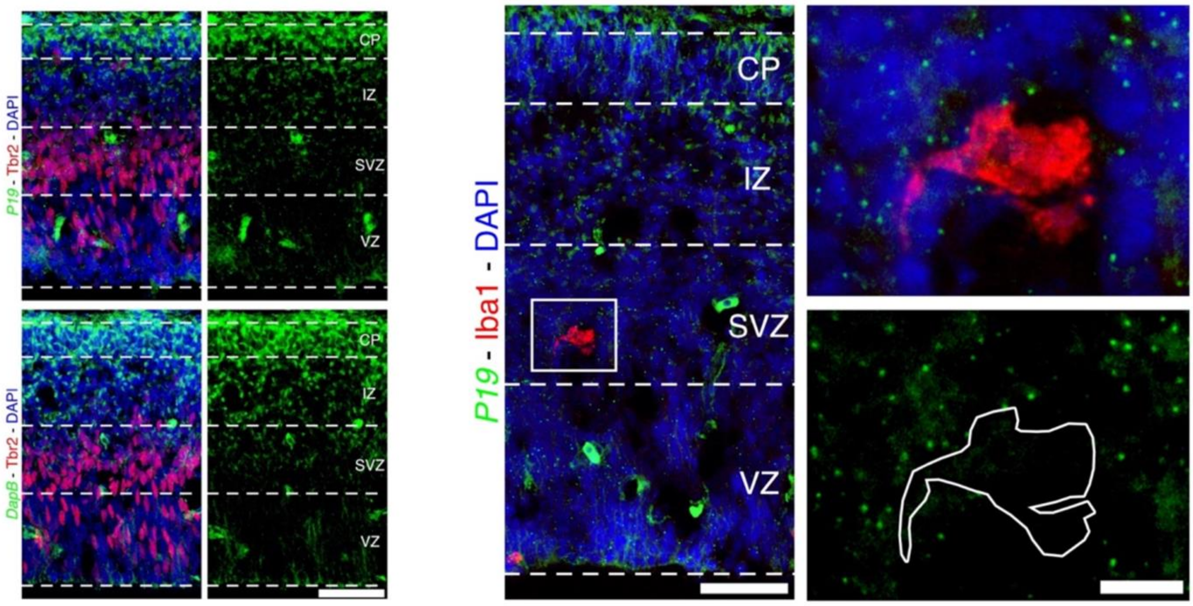

© 2023. Published by The Company of Biologists under the terms of the Creative Commons Attribution License (https://creativecommons.org/licenses/by/4.0/).

***Figure R4**. P19 in situ hybridization (green) in combination with Tbr2 (left) or Iba1 (right) immunolabeling (red). Scale bar 50 µm (low magnifications), and 10 µm (high magnification).*

We additionally invite this reviewer to see our comments to Reviewer 1, point 1 concerning expression of P19 in different cell lines and HEK293 used in our study as well as different organs and including human cells as assessed by single-cell transcriptome data from other groups.

Reviewer 2, Point 2.
If the authors are observing increased Iba1+ microglia in the VZ/SVZ but not a loss of microglia in the IZ, where are they coming from and are these indeed microglia or infiltrating macrophages? To address these questions, the authors need to evaluate microglia numbers across various regions of the cortex to see if these cells are lost somewhere else at the expense of this accumulation. Also, the authors need to use microglia specific markers (e.g., P2ry12, Fcrls) to show that these are indeed microglia, as Iba1 also marks macrophages. Similarly, the authors should use some macrophage markers (e.g., Ccr2, etc.) to show that these are not macrophages that have infiltrated the cortex. The authors should also look at Rosin and Kurrasch 2018 Journal of Neuroinflammation (impact of IUE on hypothalamic microglia) to see if IUE itself in the cortex is having any impact on microglia.

Authors reply
We thank the reviewer for raising our attention to the need of distinguishing microglia from macrophages. This comment was particularly insightful and substantially improved the quality and significance of our study. As suggested, considering that Iba1 does not distinguish between microglia and macrophages, we used P2ry12, Ccr2 as well as CD206, Lyve1 and CD68 as markers whose combinatorial expression allows the discrimination of various macrophage types including: i) microglia, ii) activated microglia, iii) non-parenchymal macrophages and iv) infiltrating, monocyte-derived macrophages. This resulted in a significantly improved dissection of P19 effects that we invite the reviewer to read in page 8 and 9, including revised Fig. 2 and S2, of our revised manuscript. In short, we found that P19 triggers the activation specifically of microglia but not other macrophage types.
With regard to the additional point raised by this reviewer in this comment, and also raised by reviewer 1 (point 4), we now investigated if Iba1+ cells migrate from not-electroporated regions adjacent to the P19 targeted area. The answer is: yes. Iba1+ cell density decreases in ventral and dorsal areas adjacent to the P19 electroporated cortical wall (see also our response to reviewer 1. point 4, and new Fig. 3G and H).

Reviewer 2, Point 3.
To understand the importance of these phenotypes (e.g., microglia accumulation & disrupted neuronal migration), the authors need to look beyond 48 hours after IUE (e.g., look at E17 & birth) to see if this is long lasting or transient. This will demonstrate the significance of the findings (i.e., transient--more of a subtle impact perhaps on connection vs. long-lasting--a prominent impact on patterning), especially with claims in the discussion about neurodevelopmental disorders and human mutations in P19 being linked to schizophrenia.

Authors reply
To address this point, we extended our analyses to brains obtained at E18 as a stage intermediate to those suggested. We found that after P19 overexpression Iba1+ cells still accumulate within the germinal zones (VZ/SVZ) (Fig. 5A-C) although to a lower extent compared to the earlier time-point analyzed. Importantly, analysis with the deep-layer neuron marker Ctip2 showed that a slower neuronal migration had long-lasting effects for the molecular identity of neurons (new Fig. 5 of the revised manuscript) strengthening our confidence about potential long-lasting neurological defects after birth.

Reviewer 2, Point 4.
The authors need to pick a better representative image for Figure 3B as it does not match what is quantified. It looks like there is a large number of microglia in the CP of RFP controls, which is much greater than what is seen in P19 (i.e., would go against the argument that P19 over-expression from HEK cells is attracting microglia if the regular RFP HEK cells are doing this). On a similar note, in control sections in Figure 2 it looks like there are almost no microglia in the CP and now in these slice experiments there appears to be a number of microglia in the area. It

© 2023. Published by The Company of Biologists under the terms of the Creative Commons Attribution License (https://creativecommons.org/licenses/by/4.0/).

is important that the authors quantify CP microglia numbers in their slices for Figure 2 and compare this to what they are getting in Figure 3 to see if the experimental method (e.g., slice culture/ex vivo preparation) itself is making microglia accumulate in the CP.

Authors reply
Please refer to our response to reviewer 1, point 5, concerning Iba1+ cell density in the RFP group. We apologize for the confusion resulting from not-representative figures, which we now corrected. Regarding the seemingly inconsistent number of microglia in the CP of brain sections as opposed to organotypic slice cultures, this has also been shown by others (Swinnen et al., 2013; Hattori et al., 2020). In essence, our results and those of others (Cunningham et al., 2013, Hattori et al., 2020) show that at E15 the CP is virtually devoid of microglia; which is colonized only after E17/E18. Nevertheless, upon culture of E14 brain slices, a fraction of the microglia/macrophage population can be observed migrating within the CP (Swinnen et al., 2013; Hattori et al., 2020). Most likely, this aspect reflects differences in vivo versus ex vivo. To improve clarity, we discuss these aspects in the results section (line 393).

Reviewer 2, Minor Comments.
For almost all of the figures (e.g., Figure 1C, Supplemental Figure 1) the authors need different coloured arrows pointing to single vs. double positive cells (there are a lack of arrows pointing to what should be focused on in the Figures).

Arrows and arrowheads with different colors have now been included to the panels, e.g. Fig. 1C, G; 2F, H; S1C, G, H; S3A, C; in addition to preexisting strategies to guide reader's attention, e.g.: continued and dashed lines (Fig. 1E) or backets (Fig. 3A).

Figure 1E, please include a lower magnification image so we can observe an appropriate area for comparison to the quantification. Please also include DAPI in these images so we can see the nuclei of the individual cells.

A lower magnification panel, including DAPI, is now shown in Supp. Fig. 1K.

For the analysis of microglia morphology in Supplemental Figure 2J, please include example images of what was considered amoeboid and what was considered ramified, in addition to including high magnification images from control and P19 over-expression animals.

Panels in Supp. Fig. 3A now contain examples of the different morphologies scored.

The authors need to show both control and P19 over-expression IUE images for Supplemental Figure 2K.

Panels in Supp. Fig. 3C now show examples of both control RFP and P19/RFP electroporated brain slices counterstained with Iba1 and EdU.

In the text (line 292) the authors refer to Figure 2M but it should be 2J.

We have corrected this Figure reference in the text, and screened the whole manuscript to avoid similar mistakes.

In the text (e.g., discussion) the authors should also include publications that discuss microglia-neural progenitor interactions outside of the cortex, as this is not a unique to the cortex (e.g., Rosin et al., 2021 Developmental Cell - microglia-progenitor interactions in the hypothalamus).

We appreciate that the Reviewer brought this study to our attention. The work by Rossin et al. is now cited together with other seminal studies highlighting the neuro-immune crosstalk during development (line 483).

CROSS-CONSULTATION COMMENTS
It looks like some of the comments that I made overlap with comments that were made by Reviewer 1, which is nice to see and demonstrates that there are key areas that need to be addressed by revising the current study/manuscript, including additional experimentation. I

© 2023. Published by The Company of Biologists under the terms of the Creative Commons Attribution License (https://creativecommons.org/licenses/by/4.0/).

also agree with the other suggestions made by Reviewer 1, and together agree that the authors will likely need 3-6 months to revise the study/manuscript.

Reviewer #2 (Significance):
The findings that Mestres and Calegari present in this manuscript are important to the microglia field as work on microglial roles during neurodevelopment (e.g., embryogenesis) are lacking--especially as it relates to how they interact with neighbouring cells to impact neurodevelopmental programs. Moreover, the authors use some very interesting and unique methodologies (e.g., live-cell imaging, ex vivo culture with cells, etc.) that will be helpful in advancing the microglia field for research conducted in the embryonic cortex.

Original submission

First decision letter

MS ID#: DEVELOP/2022/201574

MS TITLE: 4931414P19Rik: A Chemoattractant Secreted by Neural Progenitors Modulates Microglia Activation and Neuronal Migration During Mammalian Brain Development

AUTHORS: Ivan Mestres and Federico Calegari

I have now received all the referees reports on the above manuscript, and have reached a decision. The referees' comments are appended below, or you can access them online: please go to BenchPress and click on the 'Manuscripts with Decisions' queue in the Author Area.

The overall evaluation is positive and we would like to publish a revised manuscript in Development, provided that the referees' comments can be satisfactorily addressed. Please attend to all of the reviewers' comments in your revised manuscript and detail them in your point-by-point response. I have also discussed your manuscript with another editor and we both agree it is necessary that you experimentally address the remaining reviewer concerns. This includes concerns about the need for validation of overexpression, as well as concerns about the new in situs. These data suggest possible confusing expression outside of the progenitor zones.

If it would be helpful, you are welcome to contact us to discuss your revision in greater detail. Please send us a point-by-point response indicating your plans for addressing the referee’s comments, and we will look over this and provide further guidance.

Reviewer 1

Advance summary and potential significance to field

Summary: Mestres and Calegari use C57BL/6J mice to demonstrate that the uncharacterized transcript 4931414P19Rik (P19 for short) is up-regulated by neurogenic progenitors during mouse corticogenesis. By over-expressing P19 during embryogenesis in the cortex using in utero electroporation, the authors demonstrate that P19 over-expression results in the accumulation of microglia near P19-expressing cells and this ultimately inhibits neuronal migration.

Significance: The findings that Mestres and Calegari present in this manuscript are important to the microglia field as work on microglial roles during neurodevelopment (e.g., embryogenesis) are lacking--especially as it relates to how they interact with neighbouring cells to impact neurodevelopmental programs. Moreover, the authors use some very interesting and unique methodologies (e.g., live-cell imaging, ex vivo culture with cells, etc.) that will be helpful in advancing the microglia field for research conducted in the embryonic cortex.

© 2023. Published by The Company of Biologists under the terms of the Creative Commons Attribution License (https://creativecommons.org/licenses/by/4.0/).

Comments for the author

Major comments:
1) There are still concerns about monitoring P19 over-expression levels (not just normal P19 expression, but P19 over-expression following IUE--qPCR, western, etc., comparing control to P19 IUE would help).
2) The in situs included in the response document raise concerns about the specificity of the signal (the RNAScope image included shows a lot of background/non-specific staining which is a major concern--I recommend speaking with the company, as I have not experienced this high level of background with RNAScope during embryogenesis), and the signal is now evident in the CP and IZ (response document-- concerning).

Minor comments:
1) The microglia field is changing the nomenclature used to discuss microglia (see Paolicelli et al., 2022 Neuron), so I would recommend reading this paper and removing the word "activation" when referring to the change in microglia seen (might want to say that microglia react to P19 by accumulating or moving to the...etc.). The word "activation" should be removed from the title (line 9), abstract (lines 43 and 44) and main text of the manuscript (lines 98, 272, 279, 351, 356, 364, 431, 449, 470, and 490).
2) I would change the word "sub-population" on line 256 to something else (e.g., cell types, because people don't often call microglia a subpopulation of macrophages--again see publication on nomenclature).

Reviewer 2

Advance summary and potential significance to field

Although more detailed mechanisms are not yet clear, this work is the first functional analysis of P19 during brain development, suggesting that neuronal progenitor cells, in terms of secreting proteins, are also regulating microglia population and activation during embyogenesis which in turn affects the corticogenesis. This is also in line with previous work showing microglia are involved in sculpturing neural network. This work would be interesting to scientists working on brain development.

Comments for the author

In the revised version, the authors have added a substantial amount of new data which addressed most of my concerns. However, the expression level of P19 in the overexpression group is still not clear. In Supp. Fig. 1., ISH was done to show the P19 mRNA in wt mice. How about in P19RFP+ cells? Ideally, the authors can perform ISH for P19 in the overexpression group and quantify the puncta in RFP+ vs RFP- cells. Also, the quality of the ISH of P19 does not look convincing. Many green cloudy signal can be seen, even in the negative control. Alternatively, qPCR can be done to compare, in total, how much P19 is expressed in the overexpression group and wt group. Since the knockdown experiments failed, it may be worth of discussing the potential “side effect” by using an overexpression system.

First revision

Author response to reviewers' comments

POINT-BY-POINT RESPONSE TO THE REVIEWERS

Authors’ replies: red text

Authors’ general statement

© 2023. Published by The Company of Biologists under the terms of the Creative Commons Attribution License (https://creativecommons.org/licenses/by/4.0/).

We much appreciate the editor and reviewers' dedication and time in evaluating our manuscript. Below, Reviewers will find a point-by-point reply to their comments including the addition of new experiments accompanied by a revised manuscript within which all changes were highlighted in red. We hope that this revised version will be to the reviewers' satisfaction and leading to a timely dissemination of our findings.

Reviewer 1 Advance Summary and Potential Significance to Field:
Summary: Mestres and Calegari use C57BL/6J mice to demonstrate that the uncharacterized transcript 4931414P19Rik (P19 for short) is up-regulated by neurogenic progenitors during mouse corticogenesis. By over-expressing P19 during embryogenesis in the cortex using in utero electroporation, the authors demonstrate that P19 over-expression results in the accumulation of microglia near P19-expressing cells and this ultimately inhibits neuronal migration.

Significance: The findings that Mestres and Calegari present in this manuscript are important to the microglia field as work on microglial roles during neurodevelopment (e.g., embryogenesis) are lacking--especially as it relates to how they interact with neighbouring cells to impact neurodevelopmental programs. Moreover, the authors use some very interesting and unique methodologies (e.g., live-cell imaging, ex vivo culture with cells, etc.) that will be helpful in advancing the microglia field for research conducted in the embryonic cortex.

Reviewer 1 Comments for the Author:

Major comments:
1) There are still concerns about monitoring P19 over-expression levels (not just normal P19 expression, but P19 over-expression following IUE--qPCR, western, etc., comparing control to P19 IUE would help).

Authors reply
We now have included a new experiment in which P19-RFP+ targeted cells were FAC-sorted 48 hours after in utero electroporation. For control, RFP+ cells were equally FAC-sorted upon electroporation with RFP control plasmids, thus ensuring that P19 expression was quantified within a comparable population of neural stem cells, progenitors and newborn neurons resulting upon electroporation. Finally, qRT-PCR was performed and P19 levels compared in the two conditions. This showed that our approach increased by ca. 3-fold the expression of P19 within targeted cells. In essence, considering that electroporation efficiency allows the targeting of about 25-30% of cells in the relevant cortical area, such a 3-fold within the targeted pool of cells implies that our approach overall resulted in about a doubling in extracellular P19 relative to physiological conditions. Results of this experiment are now provided at line 127 of the revised manuscript.

Reviewer 1 Comments for the Author:
2) The in situs included in the response document raise concerns about the specificity of the signal (the RNAScope image included shows a lot of background/non-specific staining which is a major concern--I recommend speaking with the company, as I have not experienced this high level of background with RNAScope during embryogenesis), and the signal is now evident in the CP and IZ (response document-- concerning).

Authors reply
We agree that the background signal, particularly in the CP, is high. However, we were unable to significanlty improve this despite extensive efforts and troubleshooting including, as suggested by this reviewer, requesting support to the company. Our efforts included: 1) the use of freshly cut tissue sections, 2) longer target retrieval treatment, 3) testing different antibody concentrations, and 4) imaging at higher magnification. In this regard, we would like to quote a comment resulting from our consultations with staff at Bio-Techne, the company where RNAscope was developed, their comment was: "*congratulations on your nice pictures*" and "*brain tissue is prone to autofluorescence*" to imply that they probably consider this within the reasonable limits of the technique.
Despite being unable to significantly improve our ISH signal-to-noise level, we are firmly convinced that this does not change in any way the quality of our study and strength of its conclusions. First, the unspecific signal is diffused, cloudy and similarly detected intra- and extra-cellularly (rather than localized as bright nuclear speckles characteristic of transcribed RNA).

© 2023. Published by The Company of Biologists under the terms of the Creative Commons Attribution License (https://creativecommons.org/licenses/by/4.0/).

Second, unspecific signal is particularly high in irrelevant brain areas such as near the meninges, within the CP, and blood vessels (as opposed to nuclei of apical and basal progenitors within the VZ/SVZ). Third, unspecific signal was also detected with the same pattern in other channels and equally observed in negative control ISH (none of which obviously applied to the specific signal). When accounting for these clear differences, quantifying physiological P19 levels in cortical cell types resulted in a clear up-regulation in basal progenitors relative to both apical progenitors and neurons.
Finally, and most importantly, we would like to remind the reviewer that these ISH were just meant to provide additional support to the above claim that P19 expression is physiologically up-regulated in basal progenitors. Independently from the technical difficulties in achieving an ideal balance between signal-to-noise ratio in our ISH, this claim was already confirmed and extended by a series of independent studies reporting single-cell transcriptome analyses of cortical cell types during development including Cao et al., ***Nature***, 2019; Telley et al., ***Science***, 2019; di Bella et al., ***Nature***, 2021). All these studies (and others not cited) confirm our claim that P19 is up-regulated in neurogenic progenitors relative to neural stem cells (from 1.5- to 2-fold, depending the study considered).

Minor comments:
1) The microglia field is changing the nomenclature used to discuss microglia (see Paolicelli et al., 2022, Neuron), so I would recommend reading this paper and removing the word "activation" when referring to the change in microglia seen (might want to say that microglia react to P19 by accumulating or moving to the...etc.). The word "activation" should be removed from the title (line 9), abstract (lines 43 and 44) and main text of the manuscript (lines 98, 272, 279, 351, 356, 364, 431, 449, 470, and 490).

We thank the reviewer for bringing up this publication to our attention, which we also now cited in our manuscript (line 281). Following this article and the updated consensus nomenclature, we changed the word “activation” for “reactivation”, which acknowledges the homeostatic “active” state of microglia, and their “reactivation” upon or “response to” changes in their environment.

2) I would change the word "sub-population" on line 256 to something else (e.g., cell types, because people don't often call microglia a subpopulation of macrophages--again see publication on nomenclature).

As suggested, we changed the word “sub-population” for “cell types”.

Reviewer 2 Advance Summary and Potential Significance to Field:
Although more detailed mechanisms are not yet clear, this work is the first functional analysis of P19 during brain development, suggesting that neuronal progenitor cells, in terms of secreting proteins, are also regulating microglia population and activation during embyogenesis which in turn affects the corticogenesis. This is also in line with previous work showing microglia are involved in sculpturing neural network. This work would be interesting to scientists working on brain development.

Reviewer 2 Comments for the Author:
In the revised version, the authors have added a substantial amount of new data which addressed most of my concerns. However, the expression level of P19 in the overexpression group is still not clear. In Supp. Fig. 1., ISH was done to show the P19 mRNA in wt mice. How about in P19RFP+ cells? Ideally, the authors can perform ISH for P19 in the overexpression group and quantify the puncta in RFP+ vs RFP- cells. Also, the quality of the ISH of P19 does not look convincing. Many green cloudy signal can be seen, even in the negative control. Alternatively, qPCR can be done to compare, in total, how much P19 is expressed in the overexpression group and wt group. Since the knockdown experiments failed, it may be worth of discussing the potential “side effect” by using an overexpression system.

Authors reply
We thank the reviewer for these valuable comments, which are in fact similar, if not identical, to those of reviewer 1. We therefore invite this reviewer to read our responses above regarding both ISH background and assessing P19 expression upon electroporation by qRT- PCR.
Regarding possible side effects of electroporation, we do agree that these are entirely possible!

© 2023. Published by The Company of Biologists under the terms of the Creative Commons Attribution License (https://creativecommons.org/licenses/by/4.0/).

However, for as much as we could assess upon comparison of un-manipulated, naïve control brains with both the targeted and contralateral side of brains electroporated with RFP control plasmids, neither electroporation itself nor RFP overexpression induced any visible microglia response or change in the density of Iba1+ cells (see graph below). We now added this information in line 252 of the revised manuscript as well as a note of caution concerning possible reactions that could have been undetected (line 460).

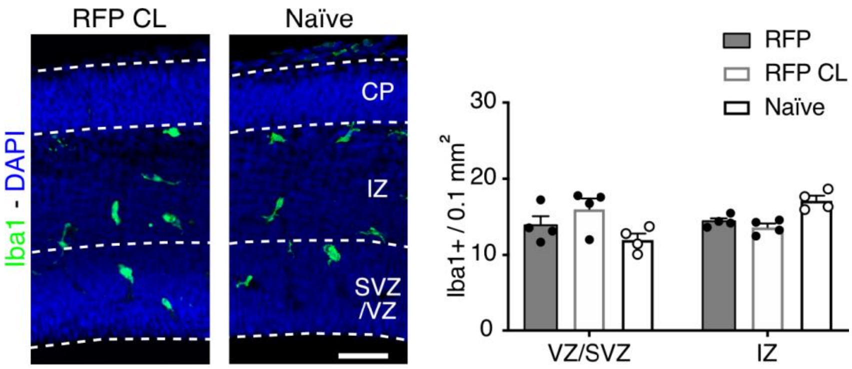

<u>Second decision letter</u>

MS ID#: DEVELOP/2022/201574

MS TITLE: 4931414P19Rik: A Chemoattractant Secreted by Neural Progenitors Modulates Microglia Activation and Neuronal Migration During Mammalian Brain Development

AUTHORS: Ivan Mestres and Federico Calegari

I have now received all the referees reports on the above manuscript, and have reached a decision. The referees' comments are appended below, or you can access them online: please go to BenchPress and click on the 'Manuscripts with Decisions' queue in the Author Area.

The overall evaluation is positive and we would like to publish a revised manuscript in Development, provided that the referees' comments can be satisfactorily addressed. Please attend to all of the reviewers' comments in your revised manuscript and detail them in your point-by-point response. In particular, in your final version, please modify the wording per reviewer 1, “Changing the word "activation" to "reactivation" throughout the manuscript is not appropriate.” I agree with reviewer 2 that the in situs of overexpression samples would be valuable, however I don't think this is necessary for publication given the other data you have presented. If you do not agree with any of their criticisms or suggestions explain clearly why this is so. If it would be helpful, you are welcome to contact us to discuss your revision in greater detail. Please send us a point-by-point response indicating your plans for addressing the referee’s comments, and we will look over this and provide further guidance.

<u>Reviewer 1</u>

Advance summary and potential significance to field

See previous summary.

Comments for the author

Minor revisions:
Changing the word "activation" to "reactivation" throughout the manuscript is not appropriate.
Reactivation = the action or process of restoring something to an active state (basically still saying the microglia are become active).

© 2023. Published by The Company of Biologists under the terms of the Creative Commons Attribution License (https://creativecommons.org/licenses/by/4.0/).

React to = an automatic instinctive unlearned reaction to a stimulus.
As I mentioned previously, the language should be changed to "microglia react to P19 by accumulating or moving to the..." or something similar.

Reviewer 2

Advance summary and potential significance to field

In this revised version, the authors performed qPCR to FACSorted RFP+ cells to quantitatively showed the overexpression of P19, which has addressed my previous concern.

Comments for the author

They also made many efforts to reduce the background noise of the ISH, though they did not really achieved the goal. I can more or less accept/understand the difficulties do exist to eliminate the ISH background. I would just suggest (not insist) the authors may perform ISH on their P19-overexpression samples in which they should be able to see more positive puncta, which then can be a 'positive control' to further verify the specificity of their ISH method.

Second revision

Author response to reviewers' comments

POINT-BY-POINT RESPONSE TO THE REVIEWERS

Authors' replies: red text

We much appreciate the editor and reviewers' dedication and time in evaluating again our manuscript. Below, they will find a point-by-point reply to their comments.

Reviewer 1 Advance Summary and Potential Significance to Field:
See previous summary.
Reviewer 1 Comments for the Author: Minor revisions:
Changing the word "activation" to "reactivation" throughout the manuscript is not appropriate.
Reactivation = the action or process of restoring something to an active state (basically still saying the microglia are become active).
React to = an automatic instinctive unlearned reaction to a stimulus.
As I mentioned previously, the language should be changed to "microglia react to P19 by accumulating or moving to the..." or something similar.

Authors reply:
We have no specific reason to prefer the word "re/activation" and are fine removing it altogether. In the current version terms like activation/reactivation/active/etc. are replaced with: "respond" or explicit description of behavior such as "accumulate", "migrate", etc. These changes are now highlighted in red (lines 35, 93, 97, 282, 290, 304, 310, 377 and 463).

Reviewer 2 Advance Summary and Potential Significance to Field:
In this revised version, the authors performed qPCR to FACSorted RFP+ cells to quantitatively showed the overexpression of P19, which has addressed my previous concern.

Reviewer 2 Comments for the Author:
They also made many efforts to reduce the background noise of the ISH, though they did not really achieved the goal. I can more or less accept/understand the difficulties do exist to eliminate the ISH background. I would just suggest (not insist) the authors may perform ISH on their P19-overexpression samples in which they should be able to see more positive puncta, which then can be a 'positive control' to further verify the specificity of their ISH method.

© 2023. Published by The Company of Biologists under the terms of the Creative Commons Attribution License (https://creativecommons.org/licenses/by/4.0/).

Authors reply:
We understand the value of additional positive controls to validate P19 expression in basal progenitors. However, we disagree that ISH in P19 electroporated cells would provide a "positive control for puncta" given that in these cells a 3-fold overexpression will now be driven from an unknown and variable number of ectopic plasmids rather than the two endogenous P19 loci within the cells' genome. It is very much expected that P19 ISH upon electroporation will result in diffused nuclear signal, rather than distinct puncta. Considering the very marginal gain of such time-consuming new experiment (even if successful, which is unlikely), and the many other independent validations of P19 expression in cortical cell type already extensively cited in our manuscript, we share the editors' opinion that this new control is not necessary.

Third decision letter

MS ID#: DEVELOP/2022/201574

MS TITLE: 4931414P19Rik: A Microglia Chemoattractant Secreted by Neural Progenitors Modulates Neuronal Migration During Corticogenesis

AUTHORS: Ivan Mestres and Federico Calegari

ARTICLE TYPE: Research Article

Thank you for sending your manuscript to Development through Review Commons

I am happy to tell you that your manuscript has been accepted for publication in Development, pending our standard ethics checks.

© 2023. Published by The Company of Biologists under the terms of the Creative Commons Attribution License (https://creativecommons.org/licenses/by/4.0/).
